# Comparative Analysis of SARS-CoV-2 Variants of Concern, Including Omicron, Highlights Their Common and Distinctive Amino Acid Substitution Patterns, Especially at the Spike ORF

**DOI:** 10.3390/v14040707

**Published:** 2022-03-29

**Authors:** Marios Nikolaidis, Athanasios Papakyriakou, Katerina Chlichlia, Panayotis Markoulatos, Stephen G. Oliver, Grigorios D. Amoutzias

**Affiliations:** 1 Bioinformatics Laboratory, Department of Biochemistry and Biotechnology, University of Thessaly, 41500 Larissa, Greece; marionik23@gmail.com; 2 Institute of Biosciences & Applications, National Centre for Scientific Research Demokritos, 15341 Agia Paraskevi, Greece; thpap@bio.demokritos.gr; 3 Laboratory of Molecular Immunology, Department of Molecular Biology and Genetics, Democritus University of Thrace, University Campus-Dragana, 68100 Alexandroupolis, Greece; achlichl@mbg.duth.gr; 4 Microbial Biotechnology-Molecular Bacteriology-Virology Laboratory, Department of Biochemistry and Biotechnology, University of Thessaly, 41500 Larissa, Greece; markoulatosp@gmail.com; 5 Department of Biochemistry, University of Cambridge, Sanger Building, 80 Tennis Court Road, Cambridge CB2 1GA, UK; sgo24@cam.ac.uk

**Keywords:** SARS-CoV-2, COVID-19, Omicron, variants of concern, evolution, spike, amino acid substitutions, recurrent mutations, dN/dS

## Abstract

In order to gain a deeper understanding of the recently emerged and highly divergent Omicron variant of concern (VoC), a study of amino acid substitution (AAS) patterns was performed and compared with those of the other four successful variants of concern (Alpha, Beta, Gamma, Delta) and one closely related variant of interest (VoI—Lambda). The Spike ORF consistently emerges as an AAS hotspot in all six lineages, but in Omicron this enrichment is significantly higher. The progenitors of each of these VoC/VoI lineages underwent positive selection in the Spike ORF. However, once they were established, their Spike ORFs have been undergoing purifying selection, despite the application of global vaccination schemes from 2021 onwards. Our analyses reject the hypothesis that the heavily mutated receptor binding domain (RBD) of the Omicron Spike was introduced via recombination from another closely related Sarbecovirus. Thus, successive point mutations appear as the most parsimonious scenario. Intriguingly, in each of the six lineages, we observed a significant number of AAS wherein the new residue is not present at any homologous site among the other known Sarbecoviruses. Such AAS should be further investigated as potential adaptations to the human host. By studying the phylogenetic distribution of AAS shared between the six lineages, we observed that the Omicron (BA.1) lineage had the highest number (8/10) of recurrent mutations.

## 1. Introduction

Since the beginning of the COVID-19 pandemic by SARS-CoV-2 in December 2019 [1,2,3], more than 9.6 million of its genome sequences have been deposited into public repositories, such as the GISAID EpiCoV database [4]. This unprecedented wealth of genomic data has provided a unique opportunity to gain a deep understanding of the patterns of viral microevolution. Signs of positive selection and adaptation are important to help us understand how different lineages with new properties emerge and spread, as an epidemic or pandemic evolves. SARS-CoV-2, like all other Coronaviruses (CoVs), possesses a replication proof-reading mechanism conferred by the nsp14 exonuclease, that significantly reduces the mutation rate to a level close to that of DNA viruses. Evolutionary analyses [5,6,7,8,9] estimate a mutation rate of 0.5 × 10^−3^–1.1 × 10^−3^ substitutions/site/year, which translates to approximately 1.3–2.8 substitution/month for the entire genome. A genome-sequencing study of samples from an immune-compromised patient who had a year-long SARS-CoV-2 infection (335 days) also reported a similar mutation rate based on the number of accumulated mutations for that period [10].

Mutations in SARS-CoV-2 are used to construct phylogenetic lineages and clades according to three nomenclature systems, PANGO, Global Initiative on Sharing All Influenza Data (GISAID), and Nextstrain [4,7,11,12,13]. The various lineages, their characteristic mutations, and their phenotypic impact are being extensively investigated and reviewed [14,15,16]. In addition, certain lineages/clades are further characterized by the World Health Organization (WHO) and the Centers for Disease Control and Prevention (CDC) as variants of interest (VoI) or concern (VoC), based on certain phenotypic criteria. VoC display increased transmissibility, and/or increased pathogenicity, and/or reduced neutralization by antibodies, and/or reduced detection by diagnostic methods [17].

Despite its relatively low mutation rate for an RNA virus, the total number of rounds of SARS-CoV-2 viral replication has been enormous, due to the absolute numbers of infections and re-infections worldwide over the last two years. Thus, the opportunity for the virus to explore its fitness landscape and better adapt to the human biology is substantial. Early molecular clock analyses have estimated that the most recent common ancestor of available SARS-CoV-2 sequences emerged between October and December 2019 [6,18,19]. Within the first year of the pandemic, SARS-CoV-2 displayed a slightly higher mutation rate than SARS-CoV-1, MERS-CoV (a beta-genus Merbecovirus) or HCoV-OC43 (a beta-genus Embecovirus); however, it did not undergo phenotypically significant mutational changes [20,21]. According to time-dependent rate variation, slightly deleterious mutations observed in the first stages of an epidemic tend to be purged in later stages [22]. The first signs of adaptive mutations that increased infectivity and viral load were observed from March to April 2020, with the emergence of the Spike D614G mutation [23,24,25,26]. The Spike receptor binding motif (RBM) N439K mutation was first identified in March 2020 in Scotland and has emerged independently in multiple lineages [27]. This mutation increases the Spike protein’s affinity for the hACE2 receptor and also facilitates evasion from antibody-mediated immunity [27].

From Autumn to Winter 2020, several highly mutated lineages emerged that displayed increased infectivity and/or immune-escape abilities and were later classified as VoC, based on their phenotypes. It has been hypothesized that such highly mutated variants may have emerged from immune-compromised patients [28]. Up until December 2021, WHO has identified five VoC (Alpha, Beta, Gamma, Delta, Omicron) and two VoI (Lambda and Mu). The Alpha VoC (PANGO lineage B.1.1.7) was first detected in the U.K. in late 2020 and displayed increased transmissibility [29,30]. The Beta VoC (PANGO lineage B.1.351) was first detected in South Africa in Autumn 2020; it became dominant in the region within weeks and displayed increased immune-escape abilities [31]. The Gamma VoC (PANGO lineage P.1) was first detected in late 2020 in Brazil, and it outcompeted other local variants by January 2021 and also displayed increased immune-escape abilities [32]. The Delta VoC (B.1.617.2) was first detected in India in late 2020, and displayed a significantly increased transmissibility and immune-escape ability [33,34,35]. This variant became the dominant lineage globally by November 2021.

Despite the global spread of the Delta VoC and its ability to outcompete other variants, there were signs that the pandemic wave was waning by Autumn 2021. However, a new variant, the Omicron VoC (B.1.1.529), was detected in South Africa and Botswana in late November 2021. Omicron has already outcompeted even the highly infectious Delta VoC, with an R0 assumed to be as high as 10 [36]. Epidemiological studies from the U.K. have reported that the Omicron re-infection rate is 5.4 times higher than that of Delta [37]. Omicron bears a significantly higher number of mutations than any other VoC (especially in the Spike gene) and is now being further classified into three sub-lineages, BA.1 (the originally designated Omicron), BA.2 (now designated as Omicron-2), and BA.3 [38]. We will refer to BA.1 as Omicron from this point on. Omicron can escape 26 out of 29 monoclonal antibodies [39] that target the highly mutated Spike receptor binding motif (RBM). Pseudovirus assays demonstrate that, compared to the reference Wuhan-Hu-1 strain, the Omicron VoC RBD binding affinity to human ACE2 is 2.4 times higher [39]. In addition, the high mutational load of Omicron has an impact [40] on the detection ability of several diagnostic tests; a characteristic Spike or nucleocapsid gene target failure pattern in RT-PCR tests is an indication for Omicron infection. Worryingly, the Omicron Spike has acquired the ability to bind to mouse ACE2 (in vitro) [39]. Thus, it could be established in rodents or other new animal reservoirs, with an elevated risk for accelerated evolution due to new host adaptations and recombination events, followed by re-introduction into humans or other animals.

Several evolutionary studies have analyzed and reviewed the mutational landscape of SARS-CoV-2 [15,19,20,21,41,42,43,44,45]. The non-synonymous to synonymous substitution rate (dN/dS) is usually employed in evolutionary analyses, in order to detect signals of negative and positive selection for specific codons, entire regions, and/or clades [46,47]. However, in this study, we adopt an approach similar to that of [42], where we investigate large trends by analyzing averages of amino acid substitutions (AAS), over entire proteins, across the five different VoC and one VoI (Lambda) that are closely related to Omicron. Our approach focuses on AAS of successful lineages that were designated as VoC, with frequencies (in any given lineage) ≥5%. Thus, we filter out many AAS of unsuccessful lineages that may have a negative or neutral effect on the virus’ fitness and are observed only in very low frequencies. In addition, by this stringent approach, we also filter out any artifactual mutations that may arise due to sequencing errors and/or dual infections. Our goal is to better understand the common and unique AAS features/patterns of the successful VoCs and use this comparative approach to gain an even deeper understanding of the recently emerged Omicron variant.

## 2. Materials and Methods

### 2.1. Detection of Amino Acid Substitutions and Calculation of Their Frequencies

For our amino acid substitution frequency analyses, we obtained from NCBI the genomes of 4 VoC (Alpha, Beta, Gamma, Delta) and one VoI (Lambda) that is closely related to Omicron. More specifically, in December 2021 we downloaded the corresponding SARS-CoV-2 data package of each of the five variant lineages by using its PANGO classification as query. A total of 1000 randomly sampled genomes were selected for each of these 4 VoCs and 679 for the Lambda VoI. A total of 136 Omicron (BA.1) and 2247 Omicron-2 (BA.2) genomes were obtained from the GISAID database. A strict quality filter was applied, whereby all genomes had a size of more than 29,400 nt, with less than 100 unsequenced nts each. The identification codes of the sequences are displayed in Supplementary File S1, spreadsheet1.

All the nucleotide sequences of each variant were separately, multiply aligned against the NCBI reference Wuhan-Hu-1 sequence (NC_045512.2) [3] by using MAFFT [48]. The 5′ and 3′ UTRs were removed from the analyses. Only nucleotide substitutions were investigated. The numbering of mutations and the coordinates of each ORF, nsp (non-structural peptides of ORF1ab) and domains/regions of interest (like the Spike RBD) was based on the genomic coordinates of the reference Wuhan-Hu-1 sequence. The frequency of each mutation within a specific variant lineage was estimated with custom-made Python scripts. We only retained nucleotide substitutions (against Wuhan-Hu-1) with a frequency of ≥5% within a given variant lineage (see Supplementary File S1, spreadsheet 2 for nucleotide changes and Supplementary File S1, spreadsheet 3 for amino acid changes). We also generated a second subset of AAS with frequencies ≥50% (within a lineage), that we call high-frequency amino acid substitutions (HF-AAS). As an extra quality control, we investigated whether the mutations detected by our analyses were also observed in the sampled GISAID sequences (of the corresponding variant lineage) at the Nextstrain webserver [4,7]. The vast majority (140/144; 97%) of HF-AAS in a certain lineage were also observed in the Nextstrain/GISAID data. Four HF-AAS were marked as nucleotide deletions (deletions that actually changed a codon). More than half (30/59; 51%) of the lower-frequency mutations (5–49% frequency in a given lineage) were also observed in the Nextstrain/GISAID webserver dataset. This may be attributed to the much lower number of representative sequences (for a particular lineage) used in the Nextstrain/GISAID webserver dataset.

### 2.2. Statistical Analyses

Statistical analyses were performed with the Python SciPy and Numpy package [49]. Visualization of graphs was performed with the Python matplotlib [50] and Biopython GenomeDiagram [51] packages.

### 2.3. dN/dS Analyses

For these analyses, only months with ≥50 high-quality sequences (for a particular VoC/VoI lineage) were included. We used 791,111 Alpha, Beta, Gamma, Delta, and Lambda sequences from NCBI and 58,837 Omicron (BA.1 and BA.2) sequences from GISAID and NCBI. More specifically, for Alpha we included 181,270 sequences of the B.1.1.7 and all Q sub-lineages. For Beta, we included 1063 sequences of the B.1.351 and its sublineages. For Gamma, we included 10,568 sequences of the P.1 and its sub-lineages. For Delta, we included 597,613 sequences of the B.1.617.2 and its AY sub-lineages. For Lambda, we included 597 sequences of the C.37 and its sub-lineages. For Omicron, we divided the analyses in the BA.1 sub-lineage (originally designated as Omicron) (56,777 sequences) and the BA.2 sub-lineage (that is now designated as Omicron-2) with 2060 sequences.

At first, we estimated the ORF1a, ORF1b, and Spike pairwise non-synonymous (dN) and synonymous (dS) rates with PAML by using the yn00 package [47,52]. We calculated the pairwise dN and dS rate of each member of each VoC/VoI lineage against the reference Wuhan-Hu-1 strain. Next, for that lineage, we calculated the average pairwise dN (avg-dN) and average pairwise dS (avg-dS) value of all its members (against the reference Wuhan-Hu-1 strain) (Supplementary File S1, spreadsheet 4).

Secondly, we reconstructed probable ancestral ORF1a, ORF1b, and Spike sequences for each VoC/VoI lineage. More specifically, for Delta, we calculated the ancestral sequence of clades 21A, 21I, and 21J separately (based on Nextclade). For Omicron, we calculated the ancestral sequence of clades BA.1 and BA.2 separately. Due to potential erroneous variant calling of available genomes, each ancestral sequence was reconstructed from the consensus sequence of the first few months of circulation of that lineage with sufficient genomes (Alpha: Nov-Dec 2020; Beta: Jan-Feb 2021; Gamma: Jan-Feb 2021; Delta 21A: Apr-May 2021; Delta 21I: Apr-May 2021; Delta 21J: Jan-Feb 2021; Lambda: Jan-Mar 2021; Omicron BA.1: Nov 2021; Omicron BA.2: Dec 2021). Next, we calculated the pairwise dN and dS values between Wuhan-Hu-1 and each of these 9 probable ancestors, for each of the three ORFs (1a, 1b, Spike) (see Supplementary File S1, spreadsheet 5). Subsequently, we also calculated how the pairwise average dN and dS values of the members of each lineage changed per month, compared to their probable lineage-ancestral sequence (see Supplementary File S1, spreadsheet 5).

As a comparison, we also generated a background dataset of ORF1a, ORF1b, and Spike sequences from non-VoC/VoI lineages. More specifically, we identified (from CoV-lineages.org) 212 non-VoC/VoI lineages with at least 500 assigned sequences (in that particular lineage). Next, for each of the 212 lineages, we randomly selected one representative sequence for each month and for each country, whenever available. This background dataset totaled 5996 sequences. We thus focused on relatively successful non-VoC/VoI lineages. We performed pairwise avg-dN and avg-dS analyses for this background dataset against the Wuhan-Hu-1 reference strain (see Supplementary File S1, spreadsheet 6).

We estimated the cumulative average synonymous and non-synonymous mutation rate of the 212 background lineages for ORF1a, ORF1b, and Spike from the beginning of the pandemic until a particular month by dividing its avg-dN and avg-dS values for that month by the number of months that had passed from the beginning of the pandemic (Wuhan-Hu-1 strain collection date: December 2019).

### 2.4. Test of the Hypothesis That the Omicron’s Highly Mutated Spike Receptor Binding Domain (RBD) Originated from Another Sarbecovirus via Recombination

The receptor binding domain (RBD) of the Omicron (BA.1) VoC has an unexpectedly high number of mutations, compared to the other four VoC. In order to account for any potential signs of recombination with an, as yet unsequenced, close relative of SARS-CoV-2, we performed the Shimodaira–Hasegawa test with CONSEL [53], for the orthologous Spike RBDs. Specifically, we used one sequence from each variant, the reference Wuhan-Hu-1 sequence, and the most closely related Sarbecoviruses from Laos. The null hypothesis constituted the accepted phylogenetic topology, with Omicron being placed within the SARS-CoV-2 clade, the Wuhan-Hu-1 sequence as basal to the other SARS-CoV-2 variants, and the Laos Sarbecovirus sequences being an outgroup. The alternative hypothesis required a tree topology where Omicron was more basal to the clade of Wuhan-Hu-1 and the other variants. The null hypothesis maximum likelihood (ML) phylogenetic trees were generated with PhyML [54] by using the SPR tree search method and the aLRT method for assessing branch support. The selected models of evolution (HKY for the nucleotide alignment and HIVw for the protein alignment) were selected by jModelTest [55] and ProtTest [56] software. The tree topology of the alternative hypothesis (Omicron being basal to the other SARS-CoV-2 sequences) was fitted (for optimizing branch lengths) with PhyML.

### 2.5. Conservation of Substituted Amino Acid Residues in Other Sarbecoviruses

As part of our analyses, we investigated whether a specific AAS of a given SARS-CoV-2 variant was observed in the homologous site of any other Sarbecovirus. Thus, for each ORF/nsp, we aligned (with MAFFT) the Wuhan-Hu-1 sequence, representative sequences from each of the 6 SARS-CoV-2 variant lineages (we used BA.1 for Omicron), 78 Sarbecovirus sequences that were analyzed by [57], and 5 Sarbecovirus sequences isolated from Laos [58] that are considered among the closest known relatives of SARS-CoV-2. Multiple alignments were manually inspected, and we only retained very well-aligned regions and sites that had an AAS in any of the 6 SARS-CoV-2 variant lineages. The aligned sites are shown in Supplementary File S2. Relative surface accessibility of selected residues that underwent mutation in at least one variant lineage and were not conserved in any other Sarbecovirus was assessed with NACCESS [59] by using available crystal structures of the nsp2, nsp3, nsp5, nsp12, nsp13, nsp14, Spike, ORF3a, Envelope, and ORF7a proteins, which were retrieved from PDBsum [60].

## 3. Results

### 3.1. Distribution and Enrichment of Amino Acid Changes in the nsps/ORFs of Each of the Six Variant Lineages Consistently Highlights the Spike ORF as an Amino Acid Substitution Hotspot

**Figure 1:**
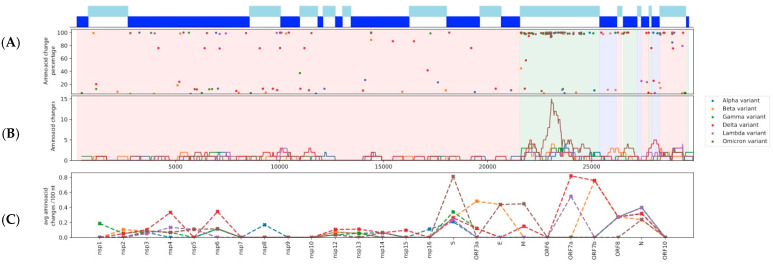
(**A**) The distribution of amino acid substitutions (AAS) across the SARS-CoV-2 genome and their frequencies for each analyzed variant lineage. (**B**) A sliding window analysis of the number of AAS for a particular region. The size of the sliding window is 500 nt with a step of 20 nt. (**C**) Number of AAS per 100 nt, for each nsp and ORF.

The various nucleotide substitutions, amino acid substitutions (AAS), their frequencies, and their absence in any VoC or other Sarbecovirus ortholog are summarized in Table 1, Figure 1 and Figure 5, in Supplementary File S1, spreadsheets 2 and 3, and in Supplementary File S2. A substantial number of cytosine nucleotide substitutions were observed and were statistically enriched, (2-fold enrichment; hypergeometric test *p*-value: 1.5 × 10^−16^), compared to the expected background. This biased mutation pattern has been ascribed to either deamination of cytosine due to the action of the host APOBEC system [61], or to methylation of CpG dinucleotides [62], or as being the result of metabolic pressure on CTP synthesis [63].

In terms of AAS, the Alpha and Lambda lineages had 24 and 25 each, the Beta and Gamma lineages had 32 and 31 each, and the Delta lineage had 43, whereas the Omicron (BA.1) lineage had 48 (Omicron-2/BA.2 had 50). Delta has a high number of AAS with frequencies (within Delta genomes) of ≥5%, which is close to those of Omicron. However, when we apply the filtering criterion of ≥50% frequency, then Delta has 28 AAS, which is still higher than Alpha, Beta, Gamma, and Lambda (15–21 AAS), but substantially lower than Omicron and Omicron-2 (45 and 49 AAS respectively). This observed pattern is probably because Delta has had more time and infections to diversify than Omicron (at the time of genome sampling). A comparison of the distributions of AAS across the genome and their frequencies in each of the six analyzed variant lineages is shown in Figure 1 and Supplementary File S1, spreadsheet 3. It is evident from Figure 1B,C that Omicron has a very distinct amino acid substitution pattern in the Spike region.

**Figure 2:**
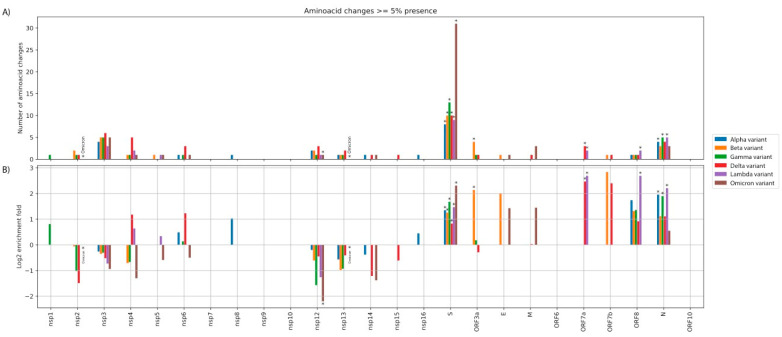
(**A**) Absolute number of amino acid substitutions (AAS) for each nsp/ORF. (**B**) Log_2_ fold enrichment of AAS for each nsp/ORF, after taking into account the length of each region. Stars denote statistically significant over/under-representation. Note that, due to the small number of AAS, several over/under-representations may not achieve statistical significance (at *p* < 0.05).

Next, we investigated, for each variant lineage independently, whether any ORFs/nsps had a statistically significant over/under-represented number of AAS, compared to the expected background. For this analysis, as background, we assumed that the AAS should be evenly dispersed across the genome.We accounted for the length of each region and performed the hypergeometric test for statistical assessment of over/under-representation. The results are shown in Figure 2. The Alpha, Beta, Gamma, Delta, Lambda, and Omicron lineages had 44%, 47%, 57%, 32%, 37%, and 67% of their HF-AAS located at the Spike, respectively, which accounts for only 12% of the genome’s length. The equivalent percentages for all AAS (≥5% frequency cut-off) were 33%, 31%, 42%, 23%, 36%, and 65% for the Alpha, Beta, Gamma, Delta, Lambda, and Omicron lineages, respectively. All variant lineages displayed a statistically significant enrichment for Spike (hypergeometric test: *p* < 0.05). Notably, the highest Spike enrichment (5-fold) is observed for the Omicron lineage. However, the Spike enrichment observed for Delta (1.8-fold) is not as high as that of the other 4 remaining variants (2.4–3.2 fold), meaning that a higher proportion of its mutations occurred outside the Spike region. When comparing the Spike AAS enrichment of Omicron to those of the other five lineages, it is always higher, and this difference is statistically significant (Fisher’s exact test *p*-value < 0.05), for all lineages except Gamma (Fisher’s exact test *p*-value < 0.065). Of note, Omicron-2 (BA.2) has 56% (28/50) of its AAS located in the Spike ORF.

We further observed that the average percentage (38–47%) of AAS located at the Spike of the six analyzed lineages (Alpha, Beta, Gamma, Delta, Lambda, Omicron—as one group) was significantly higher than the average percentage (24%) of AAS observed within the Spike of the background representative genomes from the 212 non-VoC/VoI lineages (as another group; t-test *p*-value < 0.05) (see Supplementary File S1, spreadsheet 7). This difference was statistically significant, even when we removed the Omicron lineage and even when we controlled for time by using, as another background, 170 representative non-VoC/VoI sequences with collection dates within the year 2021 (see Supplementary File S1, spreadsheet 7).

We also observed a statistically significant enrichment of AAS in the nucleocapsid of 3 lineages (Alpha, Gamma, Lambda) and an under-representation of AAS in the accessory ORFs of the Omicron (BA.1) lineage (for details, see Supplementary File S2).

### 3.2. Positive Selection Affected the Emergence of AAS in the Spike ORF of Each Variant of Concern, Followed by Purifying Selection

In all six lineages, compared to the Wuhan-Hu-1 reference strain, the non-synonymous substitutions (ranging from 27–52) were 2–3.7 times higher than the number of synonymous substitutions (ranging from 10–17), for the entire genome. In order to account for transition/transversion rate bias and base/codon frequency bias, the pairwise avg-dN/avg-dS ratio was calculated against the reference Wuhan-Hu-1 sequence, based on PAML and the Yang and Nielsen model [47,52]. For this pairwise avg-dN/avg-dS analysis, we focused on the ORF1a, ORF1b, and Spike ORF. The results are summarized in Table 1, and Supplementary File S1, spreadsheet 4. For all 6 variant lineages, the Spike avg-dN/avg-dS rate ratio ranges from 1.2–6.53. The equivalent avg-dN/avg-dS ratios for ORF1a and ORF1b are below 1 for all lineages except ORF1b of Delta 21I (1.59) and Delta 21J (1.74).

**Figure 3:**
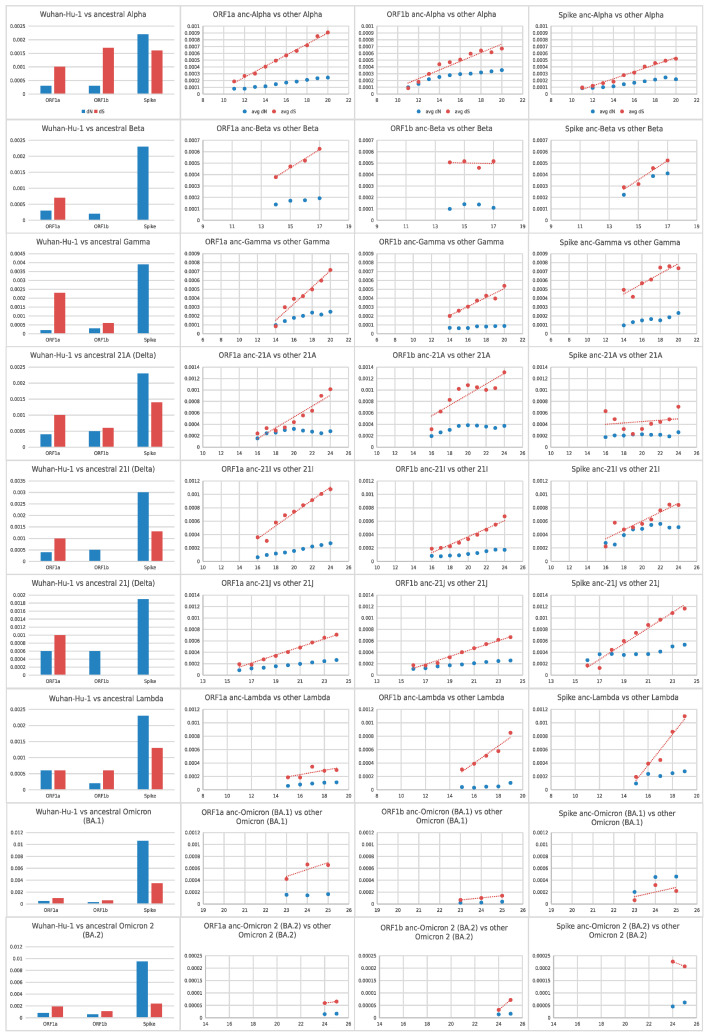
Cumulative average pairwise dN and dS values (y-axis values) of the selected variant lineages, from the beginning of the pandemic (Wuhan-Hu-1) until the ancestor of each lineage (leftmost bar-chart) and from the ancestor of each lineage until every selected month, for ORF1a, ORF1b and Spike. The x-axis of the three rightmost graphs for each lineage denotes the month from the beginning of the pandemic (December 2019). Red dots denote pairwise dS values whereas blue dots denote pairwise dN values.

Next, we reconstructed the probable ancestral ORF1a, ORF1b, and Spike sequences of each lineage separately (anc-Alpha, anc-Beta, anc-Gamma, anc-Delta_21A, anc-Delta_21I, anc-Delta_21J, anc-Lambda, anc-Omicron_BA.1, anc-Omicron2_BA.2). Based on these probable ancestral sequences, we estimated their pairwise dN and dS values against Wuhan-Hu-1 (for each of the three ORFs separately; see Figure 3). We observed that for Spike, all the progenitors of the analyzed lineages underwent positive selection, from the beginning of the pandemic (December 2019) up until the emergence of that lineage. For ORF1a, all the progenitors underwent purifying or neutral evolution. For ORF1b, the progenitors of Beta, Delta_21I, and Delta_21J lineages underwent positive selection. However, we also observed that, once a specific lineage was established, all three ORFs (1a, 1b, and Spike) have been undergoing purifying selection, with the exception of Omicron_BA.1 Spike (see Figure 3). Based on linear regression of the monthly pairwise average dS value (against the estimated ancestral sequence), we estimated the time of emergence of each probable ancestral sequence (see Supplementary File S1, spreadsheet 5). Our interpretation of all the above observations is that the progenitors of these lineages underwent positive selection, at the Spike ORF, at some point from the origin of the pandemic (December 2019) until the emergence of each lineage. Once the VoC/VoI lineage was established and started expanding, its sequences underwent purifying or neutral selection (except Omicron_BA.1), despite the global vaccination schemes.

**Figure 4:**
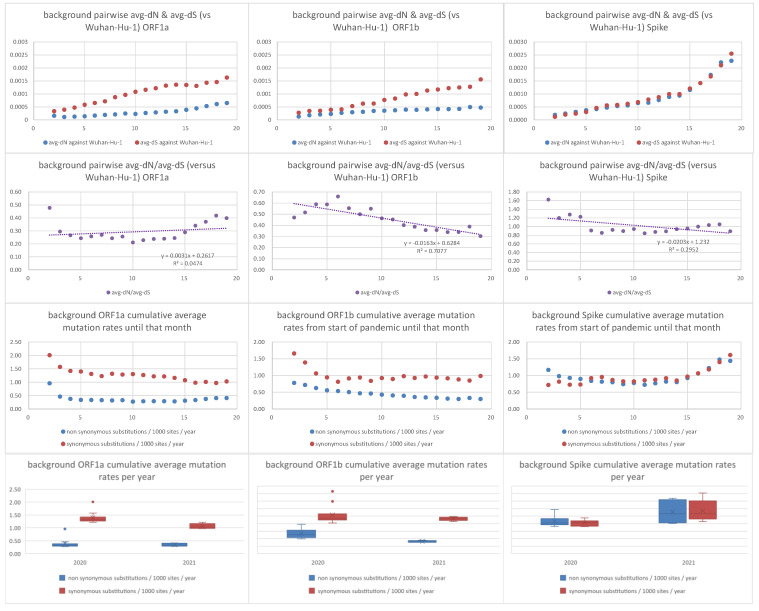
Pairwise average dN, dS, dN/dS, synonymous and non-synonymous mutation rates of background non-VoC/VoI lineages against Wuhan-Hu-1 strain. The x-axis in the first nine graphs denotes number of months from the beginning of the pandemic (December 2019).

As a comparison, we also observed how the ORF1a, ORF1b, and Spike pairwise avg-dN and avg-dS values of non-VoC/VoI lineages (against the reference Wuhan-Hu-1 strain) progressed every month. We thus obtained a dataset of 5996 sequences from 212 non-VoC/VoI lineages that constituted this background dataset. Contrary to the VoC/VoI lineages, the avg-dN/avg-dS ratio of non-VoC/VoI is very close to 1, for every month (see Figure 4 and Supplementary File S1, spreadsheet 6). Interestingly, we also observed that only for Spike (and not for ORF1a and ORF1b), both the cumulative average synonymous and non-synonymous mutation rates of non-VoC/VoI lineages increase in the second year of the pandemic (year 2021), compared to the first (year 2020).

We tested whether the Spike pairwise (against Wuhan-Hu-1) avg-dN and avg-dS rates of each of the six VoC/VoI lineages was significantly higher or significantly lower (Mann–Whitney and Student’s *t*-test, equal variance and Student’s *t*-test, unequal variance; *p*-value threshold < 0.05) than the background 212 non-VoC/VoI lineages, for each month with sufficient data (see Supplementary File S2). We observed that the monthly pairwise avg-dN (against Wuhan-Hu-1) of each of the five VoC/VoI lineages (except Omicron, where the background data are not sufficient for these months) is significantly higher than that of the background lineages. For avg-dS, the trends are not consistent.

### 3.3. The Omicron Spike-RBD Is Highly Mutated and Probably Diverged by Successive Point Mutations, Rather than by Recombination with Another Sarbecovirus

**Figure 5:**
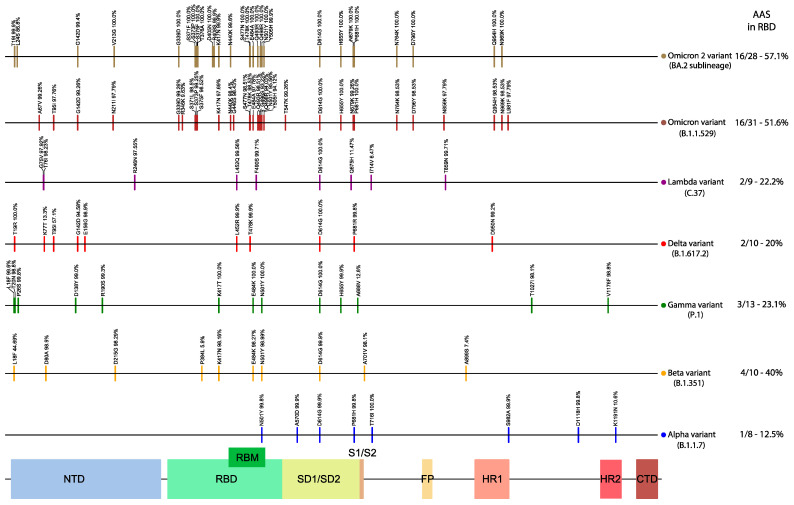
Amino acid substitutions (AAS) of the selected variant lineages (compared to Wuhan-Hu-1), across the Spike. The observed frequency of each AAS for that lineage is also displayed above the corresponding vertical bar. On the right side is the number of AAS in RBD and Table 1 sequence. NTD: N-terminal domain; RBD: receptor-binding domain; RBM: receptor-binding motif.

A very large number of the Omicron (16/31–52%) and Omicron-2 (16/28–57%) Spike-located AAS were concentrated at the RBD, whereas this was not the case for the other variant lineages (Alpha: 1/8–13%; Beta: 4/10–40%; Gamma: 3/13–23%; Delta: 2/10–20%; Lambda: 2/9–22%) (see Figure 5).

**Figure 6:**
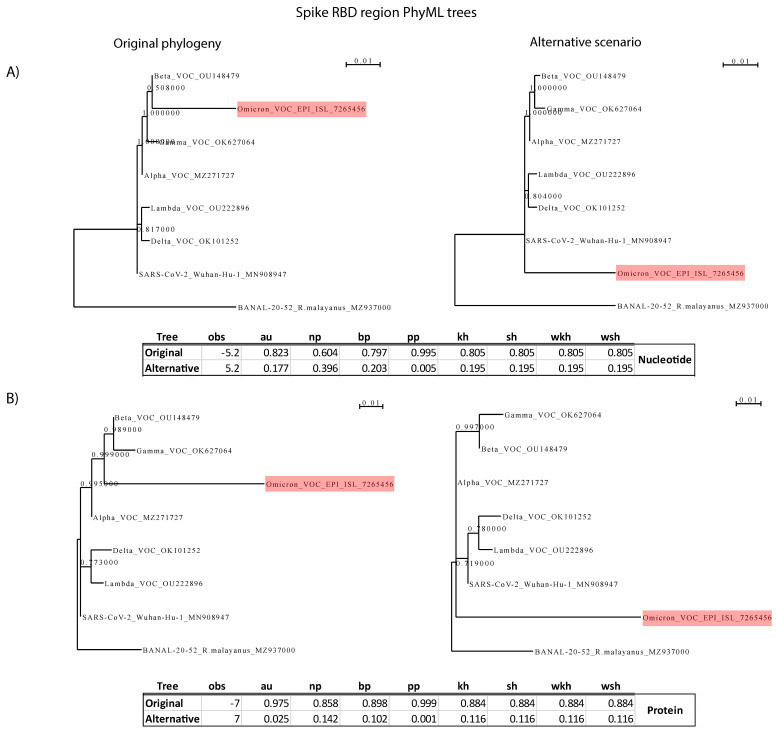
CONSEL analysis for the Spike RBD. (**A**) Analysis based on RBD nucleotide sequences. (**B**). Analysis based on RBD protein sequences. On the left side is the null hypothesis of RBD divergence by accumulation of point mutations of an existing SARS-CoV-2 lineage; on the right is Scheme 2. The branch lengths of the alternative hypothesis tree were optimized by PhML. No analysis favors the alternative hypothesis of recombination with a closely related Sarbecovirus.

Many analyses at the species, subgenus, and genus level have clearly demonstrated that Coronavirus Spike ORFs constitute intratypic and intertypic recombination hotspots [20,57,64,65,66,67,68,69,70,71]. Based on the observed high number of AAS, especially at the Spike RBD of Omicron, we tested the hypothesis that this region was introduced to the Omicron progenitor by recombination with an as yet undiscovered close relative of SARS-CoV-2. So far, the progenitor of SARS-CoV-2 remains unknown, whereas some of the currently available closest relatives (from bat hosts) shared a common ancestor approximately 40 years ago [57]. We thus performed statistical tests with CONSEL [53], in which the alternative hypothesis required that the Omicron RBD was introduced by a virus that was basal to the reference Wuhan-Hu-1 strain and other SARS-CoV-2 lineages (see Figure 6). These analyses were performed for nucleotide as well as protein sequences. The CONSEL analyses rejected the alternative hypothesis of RBD introduction from a non-SARS-CoV-2 genome. Therefore, we conclude that the high number of amino acid changes of the Omicron RBD probably emerged by accumulation of point mutations of an existing SARS-CoV-2 lineage. It should be noted that our analyses do not test for a more complex scenario, where the Omicron RBD changed by successive recombination shuffling and overprinting among different (possibly non-sequenced) SARS-CoV-2 variants. It only rejects the hypothesis of recombination with another closely related Sarbecovirus.

### 3.4. Many Amino Acid Substitutions of SARS-CoV-2 Variants of Concern Are Not Observed in Any Other Sarbecoviruses

As a proxy for the functional consequences of the observed AAS in the six variant lineages, we investigated their evolutionary conservation. More specifically, for each nsp/ORF, we generated protein multiple sequence alignments of representative sequences from each of the six variant lineages and available sequences from other Sarbecoviruses, obtained by [57,58]. We manually inspected the multiple alignments and only focused on sites that were very well aligned. We considered a variant lineage AAS as of potentially high evolutionary significance if it had a frequency of ≥50% (in that particular lineage) and if this mutated amino acid was not observed in any of the other homologous sites from the other 83 Sarbecoviruses (outside the SARS-CoV-2 lineages). The results are summarized in Table 1 and in Supplementary File S2. This analysis investigates highly conserved regions in all Sarbecoviruses (that align very well); thus it does not include more divergent regions (at the entire Sarbecovirus level), like certain fast-evolving regions within the Spike. When considering all five VoC and the Lambda VoI together, 69/109 (63%) HF-AAS were not observed in any other Sarbecovirus. More specifically, we observed 12/16 (75%), 10/15 (67%), 14/21 (67%), 19/28 (68%), 15/19 (79%), and 29/45 (64%) such AAS for the Alpha, Beta, Gamma, Delta, Lambda, and Omicron variant lineages, respectively. Of note, many amino acid changes of Omicron are situated within the fast-evolving regions of Spike that were excluded from this analysis. The relative surface accessibility (RSA) of these residues (values available for 45/69 residues) compared to the average RSA for that particular protein was, on average, 1.4-fold higher (Student’s t-test *p*-value: 1 × 10^−5^), suggesting that many of these residues are more accessible than expected by chance.

We manually investigated the frequency trends of these 69 mutations for each lineage separately in Nextstrain/GISAID, but we did not observe any notable decreasing trend over time. Of note, the Nextstrain/GISAID webserver displays a sample of ~3400 total SARS-CoV-2 sequences; thus, frequency trends for certain lineages are based on a small number of samples.

We also observed an AAS of potential interest (P132H) in the nsp5 3CL-protease of the Omicron VoC. This protease is targeted by the SARS-CoV-2-focused protease inhibitor Paxlovid, which binds at the enzyme’s catalytic site [72]. Thus, we investigated by structural simulations whether this Omicron mutation could affect either the binding of the drug or the homodimerization of the protease. This mutation is far from the catalytic site and the homodimerization surface; accordingly, these structural simulations did not demonstrate any significant effect (see Supplementary File S2). In addition, we did not observe any AAS in any of the 49 amino acids of nsp5 that are involved in the protease catalytic site, substrate binding, or the homodimerization interface, for any of the six analyzed variant lineages. This analysis was performed on the Nextstrain/GISAID webserver (8 January 2022). It should be noted that mutations and resistance to this new drug might arise in the future, as has happened for a similar HIV-protease inhibitor, ritonavir, when it was administered as a monotherapy to HIV patients [73].

### 3.5. Recurrence of High Frequency AAS in More than One VoC Lineages

A mutation may appear as recurrent, either due to positively selected point mutations for that specific amino acid (that confers some fitness advantage), by recombination, or by mistaken genome assembly/base calling of mixed infections. In the latter case, such artifactual homoplasy events are expected to be of low frequency. We thus investigated how many of the 109 HF-AAS (≥50% for a certain lineage) observed in this study were shared by two or more of the six variant lineages, and if they were recurrent in our study or in any of two previous studies [19,44]. The first study [19] identified 198 homoplasies by analyzing 7666 SARS-CoV-2 genomes from many different lineages, obtained until April 2020. That study did not include any of the six analyzed variants of this study. The more recent study of [44] analyzed more than 233,000 high quality SARS-CoV-2 genomes that were available up to January 2021. Within that second set, there were sequences from the Alpha, Beta, and Gamma lineages, as well as sequences from many other lineages. The second study observed more than 100 recurrent amino acid substitutions, with 22 of them being short-listed as strongly selected.

In our analysis, fifteen of the 109 HF-AAS were shared by two or more of the six variant lineages (see Table 2 and Supplementary File S1 spreadsheets 2 and 3). In order to determine if such events were recurrent (homoplasy/convergent evolution) mutations, or were inherited from a common ancestor (of the six VoC/VoI lineages), we investigated the distribution of each of these mutations in the Nextstrain phylogenetic tree that was constructed from more than 3400 representative sequences from various clades/lineages (see Supplementary File S2). Ten of the fifteen mutations were homoplasy events, with two of them at ORF1ab (nsp3 and nsp4), seven at the Spike ORF, and one at the nucleocapsid. Two of the five inherited mutations (nsp12:P323L; Spike:D614G) were present in all six lineages and were previously designated as recurrent [19,44], because they have reoccurred in various lineages. Another two inherited mutations (nucleocapsid:R203K; nucleocapsid:G204R) were present at the common ancestor of the Alpha, Gamma, Lambda, and Omicron lineages and were previously designated as recurrent [19,44], because they have reoccurred in various lineages. By focusing only on high-frequency mutations, we observed that the Omicron lineage had the highest number (8/10) of recurrent mutations in our analysis. Thus, it is conceivable that positive selection or/and intra-SARS-CoV-2 recombination events among known and unknown lineages may have played a significant role in the emergence of the Omicron lineage.

## 4. Discussion

This study compared the amino acid substitution (AAS) patterns of five VoC lineages (Alpha, Beta, Gamma, Delta, Omicron) and one VoI lineage (Lambda) that are relatively close to Omicron (based on the Nextstrain phylogenetic trees). The frequency of an AAS was calculated based on 1000, 679, and 136 randomly sampled sequences, for each of the four VoC (Alpha–Delta), for the Lambda VoI, and for the Omicron VoC, respectively. We focused on the highly successful VoCs instead of pooling sequences from all lineages together and cataloguing all mutations. Furthermore, by analyzing each successful lineage separately (instead of pooling all available sequences), we controlled for biases caused by oversampling of a highly successful lineage in a certain geographic region and time. A substantial number of SARS-CoV-2 genomes have been provided by genome centers in the U.K. and U.S.A., whereas sequencing efforts have been intensified worldwide at around the same time that the Delta variant dominated. As a quality control, the mutations that we detected (especially the high frequency ones, ≥50%) were further validated by Nextstrain/GISAID.

The Spike ORF constitutes 12% of the genome, but compared to the other genomic regions it consistently (for each of the six lineages) accumulates AAS much more frequently than expected by chance, assuming (as the expected background) an even distribution of AAS across the genome. These observations are in accordance with experimental evolution studies that demonstrate a 4–5-fold higher mutation rate for the Spike, compared to the rest of the genome [41]. The Omicron lineage demonstrates the highest enrichment by far, where 65–67% of its total AAS are situated within the Spike, whereas, for the other five variant lineages, the equivalent percentages range from 23–57%. This difference between Omicron and the other five variants is significant. We also observed that the six analyzed VoC/VoI lineages had a significantly higher percentage of AAS located at the Spike, compared to a background dataset of other non-VoC/VoI lineages. In addition, we observed ten high-frequency mutations that were shared by two or more of the 6 VoC/VoI-analyzed lineages and were examples of convergent evolution (homoplasies); they were not inherited from a common ancestor. Seven of them were located at the Spike ORF. Interestingly, the highest number of such homoplasy events are observed in the Omicron lineage. Whether this is due to positively selected point mutations or complex intra-SARS-CoV-2 recombination events/overprinting is very difficult to discern at this point. Homoplasies/convergent evolution has been reported in previous analyses of SARS-CoV-2 genomes [19,44,74] and has been observed in SARS-CoV-2 variants adapting to minks, with the most notable case being the Spike Y453F mutation [75].

Given the very high number of AAS within the Omicron’s Spike and especially its RBD, we considered the possibility that this region might have been acquired by intratypic homologous recombination from another closely related (non-SARS-CoV-2) Sarbecovirus. The Spike of many CoVs is a hotspot for intratypic and intertypic recombination events [20,57,64,65,66,67,68,69,70,71]. However, the CONSEL analyses of our study reject this specific evolutionary hypothesis. Similarly, another analysis rejected the hypothesis that the original/ancestral SARS-CoV-2 genome was a recombinant among any of the known Sarbecoviruses [20].

In all six analyzed lineages, the majority of high frequency AAS (≥50%) found across the genome were not observed in any other known Sarbecovirus, thus pointing either towards antigenic shift, and/or adaptation to the human host, and/or attenuation that may have allowed for sustained high infection rates. The SARS-CoV-1 sequence was included in our analyses; however, that virus only caused a limited number of human infections, and it did not have sufficient time and opportunity to adapt to the human biology, as SARS-CoV-2 did. It is also conceivable that these specific mutations may be slightly deleterious ones that “hitchhiked” on other beneficial mutations in the first stages of the SARS-CoV-2 evolution and became fixed due to high expansion. Such deleterious mutations are expected to be expunged in later stages of the virus’ evolution, according to time-dependent rate variation [22]. However, we have not observed such decreasing frequency trends so far. Experimental mutation studies can answer these important questions concerning the functional significance of these particular mutations.

An evolutionary analysis of 133,000 SARS-CoV-2 genomes sampled during the first year of the pandemic (December 2019–October 2020) revealed that the virus was evolving relatively slowly with no evidence of major increases in selective pressures [21]. Our observations/conclusions from pairwise (against Wuhan-Hu-1) dN/dS analyses of the Spike ORF from 212 non-VoC/VoI background lineages is in agreement with the above study. Interestingly, for only the Spike ORF (and not for ORF1a and ORF1b), the cumulative average synonymous and non-synonymous mutation rates increase in the second year (2021) of the pandemic, but the value of the avg-dN/avg-dS ratio still remains close to 1. In simple terms, in the second year of the pandemic, the Spike ORFs of non-VoC/VoI background strains (as a whole) seem to accumulate both synonymous and non-synonymous mutations more rapidly than in the first year. We hypothesize that this increase may be attributed to a larger diversity of sequences that may have given rise to even more diverse lineages via undetected intra-SARS-CoV-2 recombinations, analogous to a positive feedback loop. It should be remembered that the Spike ORF is a recombination hotspot. If we had observed a significant increase only in the cumulative average non-synonymous (but not synonymous) rate of the Spike or of the other two regions (ORF1a, ORF1b), then it would be reasonable to assume that such an increase could possibly be attributed to positive selection, instead of intra-SARS-CoV-2 recombination of ever more diverging lineages.

In this study, we focused on the phylogenetic lineages of six successful VoC/VoI that present significant evolutionary leaps in terms of infectivity and immune escape. Each of these lineages displays a pairwise avg-dN/avg-dS ratio above 1 for the Spike ORF. Accordingly, a recent evolutionary study identified 16 (of the 30) Omicron Spike AAS to have been evolving under positive selection [38]. In our study, the Spike ORF of the six VoC/VoI lineages appears to have undergone positive selection for some period from the beginning of the pandemic (December 2019) until their establishment. Our observation is in accordance with another recent study of four VoC (Alpha–Delta) that was based on phylogenetic/molecular clock analyses and demonstrated an episodic increase in the substitution rate of VoC progenitors [9]. Another study that investigated signs of positive selection at the codon level of several lineages identified a large number of codons that underwent adaptive evolution for some period from the beginning of the pandemic until March 2021 [74]. The number of positively selected codons significantly increased after November 2020 [74]. Our study demonstrates that once each of these particular lineages is established, its Spike undergoes neutral or purifying selection. Importantly, our analyses of the pairwise avg-dN and avg-dS values study entire regions and not specific codons; they are based on large numbers of sequences and should be robust to erroneous sequencing variant-calling. They are also complementary to phylogenetic/molecular clock or codon-wise selection analyses [9,74].

Our observations do not support a scenario where vaccinations significantly accelerate the evolution (and especially the non-synonymous substitutions) of the entire Spike ORF of already established, successful, and widely spread lineages in a stepwise manner. Whether such vaccination schemes put pressure for the emergence of new and highly divergent lineages is not clear yet. It is also conceivable that vaccination schemes may put pressure on specific codons, that our gene-wise analyses cannot detect. Each of the five major VoCs was not derived by a few extra mutation steps of another pre-existing VoC. This is possibly because steady-stepwise evolution may not be sufficient to achieve immune escape during the evolution of a pandemic where the vast majority of the population is no longer antigenically naïve (especially for the Spike ORF), but saltatory evolution may be necessary [76]. Manual inspection of the Nextstrain/GISAID phylogenetic tree shows that most of these successful lineages are deep branches, close to the root. Although the data are insufficient to draw safe conclusions yet, it is conceivable that “under-the-radar” lineage evolution may be occurring, involving a combination of host-switching and chronic infections of immune-compromised patients.

The Omicron variant demonstrates a previously unseen high infection and re-infection rate. The Wuhan-Hu-1 strain had an estimated R0 of 2.7, the Alpha lineage had 40–90% increased infectivity, and the Delta lineage was even more infectious (R0 estimated between 5–7), whereas the Omicron VoC is the most infectious so far, with an R0 assumed as high as 10 [29,30,36]. In addition, the Omicron lineage demonstrates a re-infection rate 5.4 times higher than that of the already highly infectious Delta lineage [37]. However, this increase in infectivity is not only due to higher affinity of the Spike RBD for the human ACE2 receptor. Pseudovirus assays demonstrate that the Omicron’s RBD binding affinity for the human ACE2 receptor is 2.4 times higher than that of the Wuhan-Hu-1 strain [39], which is lower than that of Alpha (6.2 times higher than Wuhan-Hu-1), similar to that of Beta (2.4 times higher than Wuhan-Hu-1), and higher than that of Delta (1.2 times higher than Wuhan-Hu-1). The high infection and re-infection rate of Omicron might be attributed, at least in part, to strong pressure to achieve antigenic shift, because it has emerged in an environment where many individuals already had been infected by other variants or were immunized to a large extent by Spike-targeting vaccines. The Omicron lineage can already escape 26 out of 29 monoclonal antibodies [39] that target the Spike RBM. It would be useful to compare SARS-CoV-2 genome sequences from populations vaccinated, not with mRNA or recombinant viruses, but with inactivated SARS-CoV-2; unfortunately, insufficient data are available to make such a comparison.

One hypothesis is that Omicron emerged within chronically infected immunocompromised patients [77,78]. Nevertheless, genome sequencing of two chronically infected immunocompromised patients did not demonstrate an amino acid substitution enrichment pattern for Spike at the level that was observed for Omicron [10,79]. More specifically, an immunocompromised patient with a year-long infection accumulated 17 amino acid substitutions after 314 days of infection, with 4 of them (24%) located at the Spike [10]. In addition, an HIV patient, by the 190th day of persistent infection accumulated 15 AAS, with five of them (33%) located at the Spike [79]. However, many more similar studies need to be performed in order to draw a safe conclusion. It is also possible that this enhanced immune-escape ability of Omicron emerged as a by-product of its progenitor adapting to another host, such as a rodent, that subsequently re-infected humans [39,45,77,78,80]. It is conceivable that, in the first phase of such a scenario, several mutations could have happened in order to adapt to that other host. In the second phase, chronic re-infection of a human host (perhaps an immunocompromised patient) may have given an opportunity for this strain to evolve epistatic mutations that compensated for the ones of the previous phase. A recent study has proposed that the Omicron’s Spike mutations individually may have a fitness cost, but they may cooperatively interact to compensate for this loss, in a positive epistatic manner [38].

Predicting the exact path of the COVID-19 pandemic is unrealistic. The phenotypic effects of even a few AAS should not be underestimated. A relatively small number of mutations is sufficient to change important properties and characteristics of a Coronavirus (CoV), such as transmissibility, replication, and even immune escape [15,23,25]. For example, a feline CoV can be transformed into a lethal form (Feline Infectious Peritonitis Virus -FIPV) by a few point mutations in the C-terminal part of the Spike ORF [81]. Such mutations change the cell-entry and tissue tropism properties of the virus from enteric epithelia to macrophages [81]. The A.30 SARS-CoV-2 lineage has demonstrated enhanced evasion from vaccine-induced antibodies and altered cell-entry properties with a preference for other cell-types that would promote extra-pulmonary spread [82]. Many different CoVs have already demonstrated a remarkable evolutionary plasticity that is achieved by point mutations, insertions/deletions, homologous intratypic, intertypic, and even non-homologous recombination (gene duplications, horizontal gene transfer) [64,83]. Based on these observations, at least five feasible evolutionary scenarios (having a range of probabilities) have been proposed [83] that may affect the path of the COVID-19 pandemic, or even the future emergence of another highly infectious CoV. The emergence of the Omicron VoC, together with the evolutionary history of the entire Coronavirus subfamily, is a strong reminder that scientists, vaccine/drug developers, and policymakers should remain vigilant. More importantly, given the inherently elevated mutation and recombination rate of the Spike ORF and the currently approved vaccination schemes and monoclonal antibody therapies that mostly target this region, other more stable genomic regions should also be extensively investigated as future targets [83,84]. Such a diversified approach would be analogous to the HIV therapeutic schemes, where combination therapies have replaced the use of single drugs, to which the virus had quickly adapted.

## Figures and Tables

**Table 1:** Summary table of the nucleotide and amino acid substitutions observed for each of the six analyzed SARS-CoV-2 variant lineages, compared to the Wuhan-Hu-1 reference sequence. Note that, for ORF8 of the Alpha variant lineage, we did not count the non-synonymous mutations that were found downstream the Q27stop mutation.

Excluding Indels	Alpha		Beta		Gamma		Delta		Lambda		Omicron (BA.1)		All VoC/VoI	
	Total	≥50%	Total	≥50%	Total	≥50%	Total	≥50%	Total	≥50%	Total	≥50%	Total	≥50%
Nucleotide substitutions	42	26	43	17	49	31	58	32	40	27	66	59	253	150
Synonymous nucleotide substitutions	14	7	10	2	17	9	14	3	13	6	14	10	74	31
Non synonymous nucleotide substitutions	28	19	32	15	32	22	44	29	27	21	52	49	178	119
Aminoacid changes	24	16	32	15	31	21	43	28	25	19	48	45	164	109
Recurrent amino acid changes in our analysis		2		3		2		5		3		8		10
Amino acid changes absent in other Sarbecoviruses	18	12	22	10	19	14	30	19	18	15	30	29	105	69
Spike nucleotide substitutions	9	8	12	7	14	12	11	10	13	9	36	35	76	63
Spike non-synonymous nucleotide substitutions	8	7	10	7	13	12	11	10	11	8	34	33	69	60
Spike synonymous nucleotide substitutions	1	1	2	0	1	0	0	0	2	1	2	2	7	3
Spike average pairwise dN/dS vs Wuhan-Hu-1	1.2		6.53		5.93		1.19–1.63		1.29		2.79		N/A	
Spike aminoacid changes	8	7	10	7	13	12	10	9	9	7	31	30	61	53
All accessory ORFs nucleotide substitutions	5	4	6	2	3	2	7	4	4	0	3	3	28	15
All accessory ORFs aminoacid changes	1	1	6	2	2	2	6	4	3	0	0	0	18	9

**Table 2:** High-frequency (≥50%) mutations shared by more than one variant lineages. Column 1 indicates whether the mutation is due to homoplasy or was inherited from a common ancestor, based on our analysis. Column 2 denotes whether that amino acid was identified as recurrent in the van Dorp et al., 2020 study [19]. Column 2 indicates whether that amino acid was identified as recurrent in the Rochman et al., 2021 study [44]. Column 4 gives the coordinates of the mutation on the Wuhan-Hu-1 reference strain. Column 5 indicates the amino acid in the Wuhan-Hu-1 reference sequence. Columns 6–11 denote the mutant amino acid and its frequency in the respective lineage. Column 12 denotes, with A, an amino acid mutation in the variant lineages that was in a highly conserved site, but absent in all other Sarbecoviruses.

Our Analysis	Recurrent van Dorp 2020	Reccurent Rochman 2021	ORF_aa_Position	Ref. aa	Alpha	Beta	Gamma	Delta	Lambda	Omicron	Sarbeco. Cons.
Homoplasy	No	No	ORF1ab_2287; nsp3_1469	P	X	X	X	S:75.43	S:97.94	X	A
Homoplasy	No	No	ORF1ab_3255; nsp4_492	T	X	X	X	I:76.13	I:99.71	I:100.0	A
Inherited	YES	YES	ORF1ab_4715; nsp12_323	P	L:99.5	L:88.69	L:99.9	L:99.7	L:99.71	L:100.0	A
Homoplasy	No	No	Spike_95	T	X	X	X	I:57.1	X	I:97.76	
Homoplasy	No	No	Spike_142	G	X	X	X	D:94.58	X	D:99.26	A
Homoplasy	No	No	Spike_417	K	X	N:98.16	T:100.0	X	X	N:97.69	A
Homoplasy	No	No	Spike_478	T	X	X	X	K:99.9	X	K:98.53	
Homoplasy	No	No	Spike_484	E	X	K:98.27	K:100.0	X	X	A:97.78	A
Homoplasy	No	YES	Spike_501	N	Y:99.8	Y:98.99	Y:100.0	X	X	Y:95.56	A
Inherited	YES	YES	Spike_614	D	G:99.9	G:99.9	G:100.0	G:100.0	G:100.0	G:100.0	A
Inherited	No	No	Spike_655	H	X	X	Y:99.9	X	X	Y:100.0	
Homoplasy	No	No	Spike_681	P	H:99.8	X	X	R:99.8	X	H:100.0	
Homoplasy	YES	No	Nucleocapsid 13	P	X	S:14.4	X	X	L:98.53	L:97.04	A
Inherited	YES	YES	Nucleocapsid 203	R	K:98.0	X	K:99.29	M:99.8	K:100.0	K:98.53	A
Inherited	YES	YES	Nucleocapsid 204	G	R:84.78	X	R:99.49	X	R:100.0	R:98.53	A

## Data Availability

Not applicable.

## Supplementary Materials

The following supporting information can be downloaded at: https://www.mdpi.com/article/10.3390/v14040707/s1. Figure S1. Alpha lineage Spike. (A) Comparison of average dN rates between the Alpha lineage and the non-VoC/VoI background lineages, for each month. (B) Comparison of average dS rates between the Alpha lineage and the non-VoC/VoI background lineages, for each month. Figure S2. Beta lineage Spike. (A) Comparison of average dN rates between the Beta lineage and the non-VoC/VoI background lineages, for each month. (B) Comparison of average dS rates between the Beta lineage and the non-VoC/VoI background lineages, for each month. Figure S3. Gamma lineage Spike. (A) Comparison of average dN rates between the Gamma lineage and the non-VoC/VoI background lineages, for each month. (B) Comparison of average dS rates between the Gamma lineage and the non-VoC/VoI background lineages, for each month. Figure S4. Delta lineage Spike. (A) Comparison of average dN rates between the Delta lineage and the non-VoC/VoI background lineages, for each month. (B) Comparison of average dS rates between the Delta lineage and the non-VoC/VoI background lineages, for each month. Figure S5. Lambda lineage Spike. (A) Comparison of average dN rates between the Lambda lineage and the non-VoC/VoI background lineages, for each month. (B) Comparison of average dS rates between the Lambda lineage and the non-VoC/VoI background lineages, for each month.
