# Supplementary material for: Comparative Analysis of SARS-CoV-2 Variants of Concern, Including Omicron, Highlights Their Common and Distinctive Amino Acid Substitution Patterns, Especially at the Spike ORF
Source: Viruses. 2022 Mar 29;14(4):707. doi: 10.3390/v14040707 (PMC9025783; doi:10.3390/v14040707)
Supplement: Supplementary file 1 [file viruses-14-00707-s001.zip › viruses-1618132-supplementary/viruses-1618132-sup/Supplementary File S2.pdf]

Supporting Information for:

Comparative analysis of SARS-CoV-2 variants of concern, including Omicron, highlights their common and distinctive amino acid substitution patterns, especially at the Spike ORF.

Marios Nikolaidis[1], Athanasios Papakyriakou[2], Katerina Chlichlia[3], Panayotis Markoulatos[4], Stephen G. Oliver[5], Grigorios D. Amoutzias[1]*

[1] Bioinformatics Laboratory, Department of Biochemistry and Biotechnology, University of Thessaly, Larissa, 41500, Greece; amoutzias@bio.uth.gr, marionik23@gmail.com
[2] Institute of Biosciences & Applications, National Centre for Scientific Research Demokritos, Agia Paraskevi, 15341, Greece; thpap@bio.demokritos.gr
[3] Laboratory of Molecular Immunology, Department of Molecular Biology and Genetics, Democritus University of Thrace, University Campus-Dragana, 68100 Alexandroupolis, Greece; achlichl@mbg.duth.gr
[4] Microbial Biotechnology-Molecular Bacteriology-Virology Laboratory, Department of Biochemistry and Biotechnology, University of Thessaly, Larissa, 41500, Greece; markoulatosp@gmail.com
[5] Department of Biochemistry, University of Cambridge, Sanger Building, 80 Tennis Court Road, Cambridge CB2 1GA, UK; sgo24@cam.ac.uk
* Correspondence: amoutzias@bio.uth.gr

Average Spike pairwise dN and dS rates per month for each of the six VoC/VoI lineages and the 212 background lineages

We investigated how the Spike ORF's pairwise average dN (avg-dN) and average dS (avg-dS) rate of each lineage (against Wuhan-Hu-1 reference strain) was changing per month (see Supplementary Figure S**1** - Supplementary Figure S**5**). All the available Spike sequences of that VoC that had collection dates of that month were compared with the reference Wuhan-Hu-1 strain. We tested whether the avg-dN and avg-dS rates of each of the six lineages was significantly higher or significantly lower (Mann-Whitney and Student's t-test, equal variance and Student's t-test, unequal variance; p-value threshold < 0.05) than the background 212 non-VoC/VoI lineages, for each month with sufficient data. We observed that the Spike monthly avg-dN of each of the five VoC/VoI lineages (except Omicron, where the background data are not sufficient for these months) is significantly higher than that of the Spike ORF of background lineages. For avg-dS, the trends are not consistent.

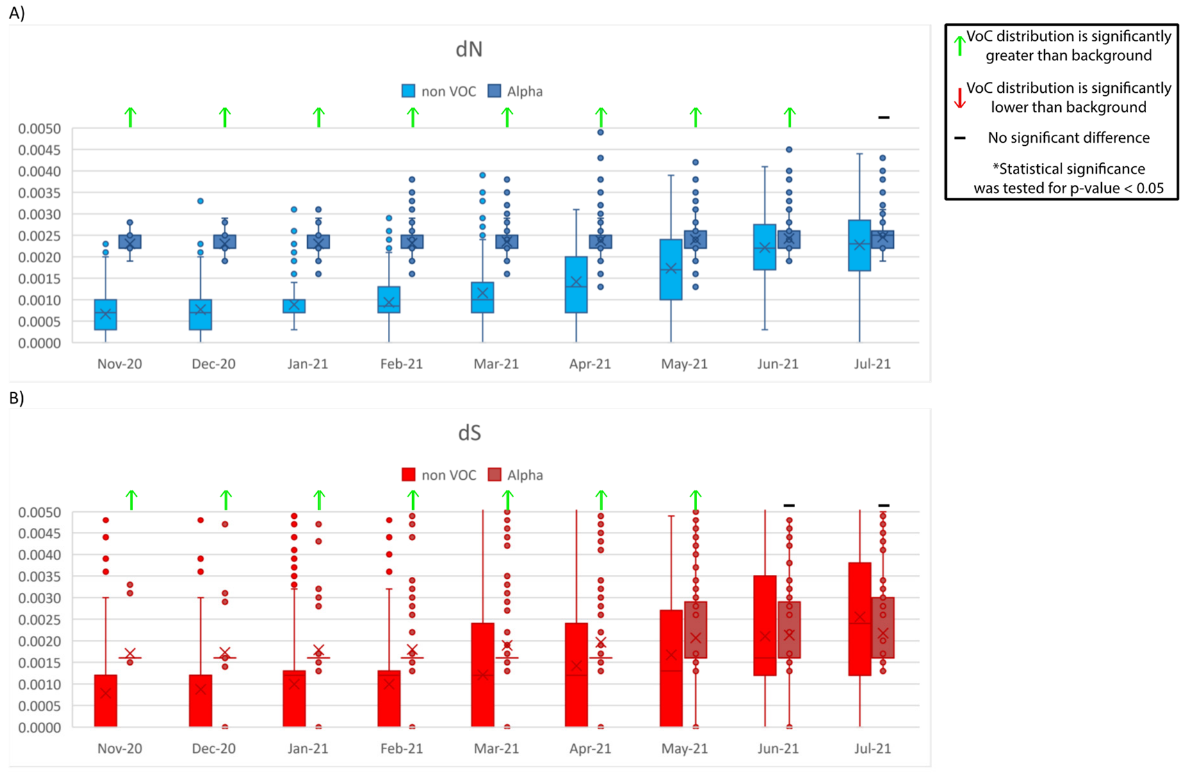

Supplementary Figure S1. Alpha lineage Spike. A) Comparison of average dN rates between the Alpha lineage and the non-VoC/VoI background lineages, for each month. B) Comparison of average dS rates between the Alpha lineage and the non-VoC/VoI background lineages, for each month.

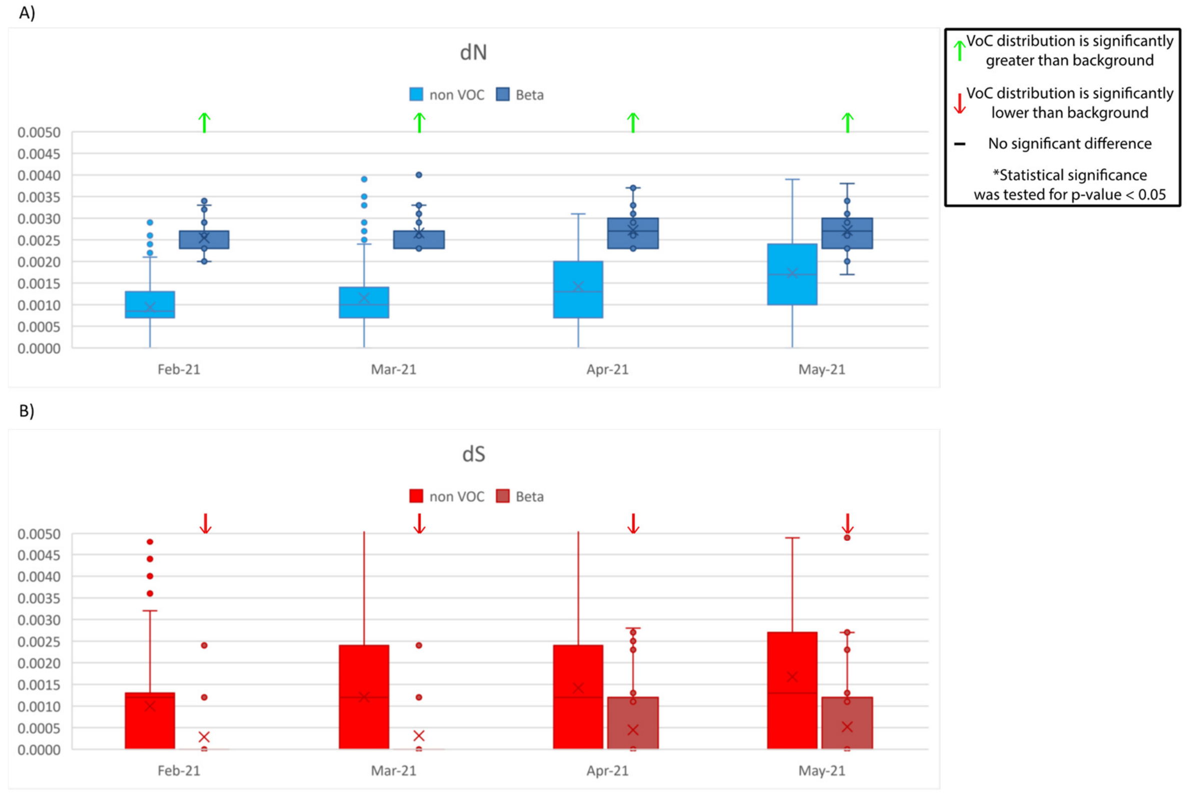

Supplementary Figure S2. Beta lineage Spike. A) Comparison of average dN rates between the Beta

lineage and the non-VoC/VoI background lineages, for each month. B) Comparison of average dS rates between the Beta lineage and the non-VoC/VoI background lineages, for each month.

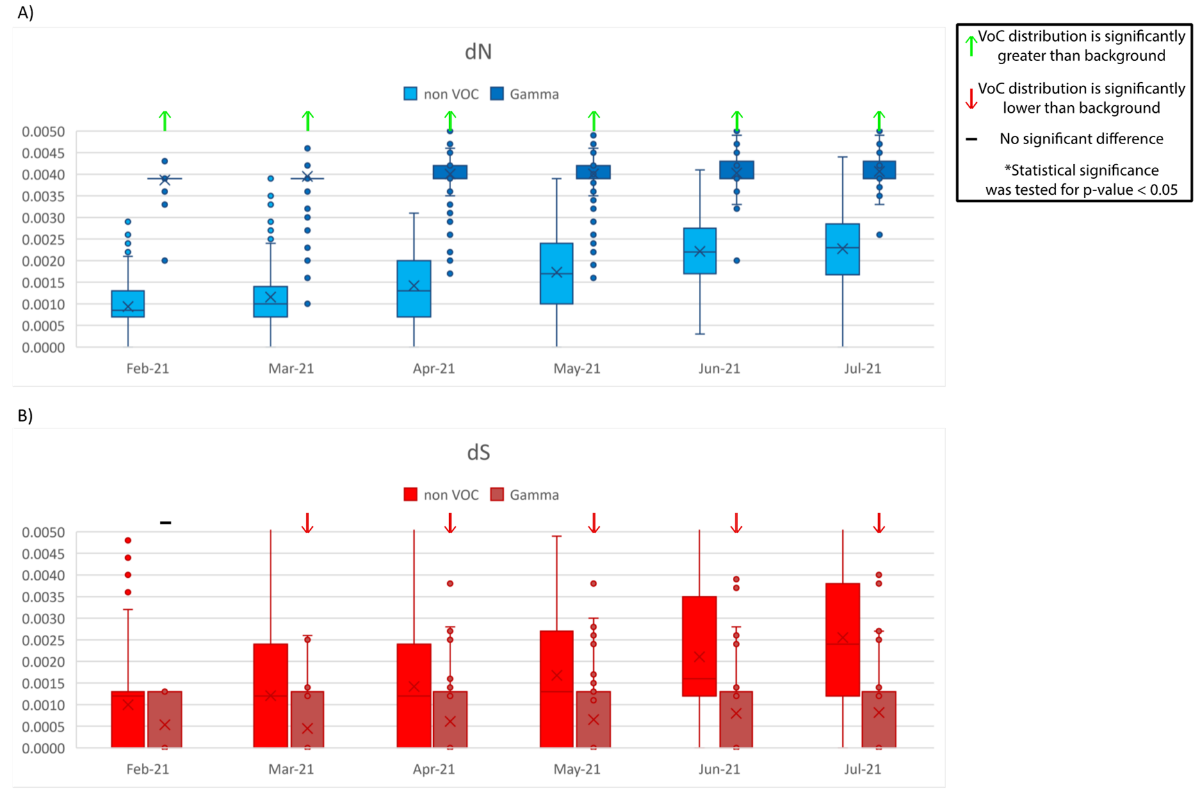

Supplementary Figure S3. Gamma lineage Spike. A) Comparison of average dN rates between the Gamma lineage and the non-VoC/VoI background lineages, for each month. B) Comparison of average dS rates between the Gamma lineage and the non-VoC/VoI background lineages, for each month.

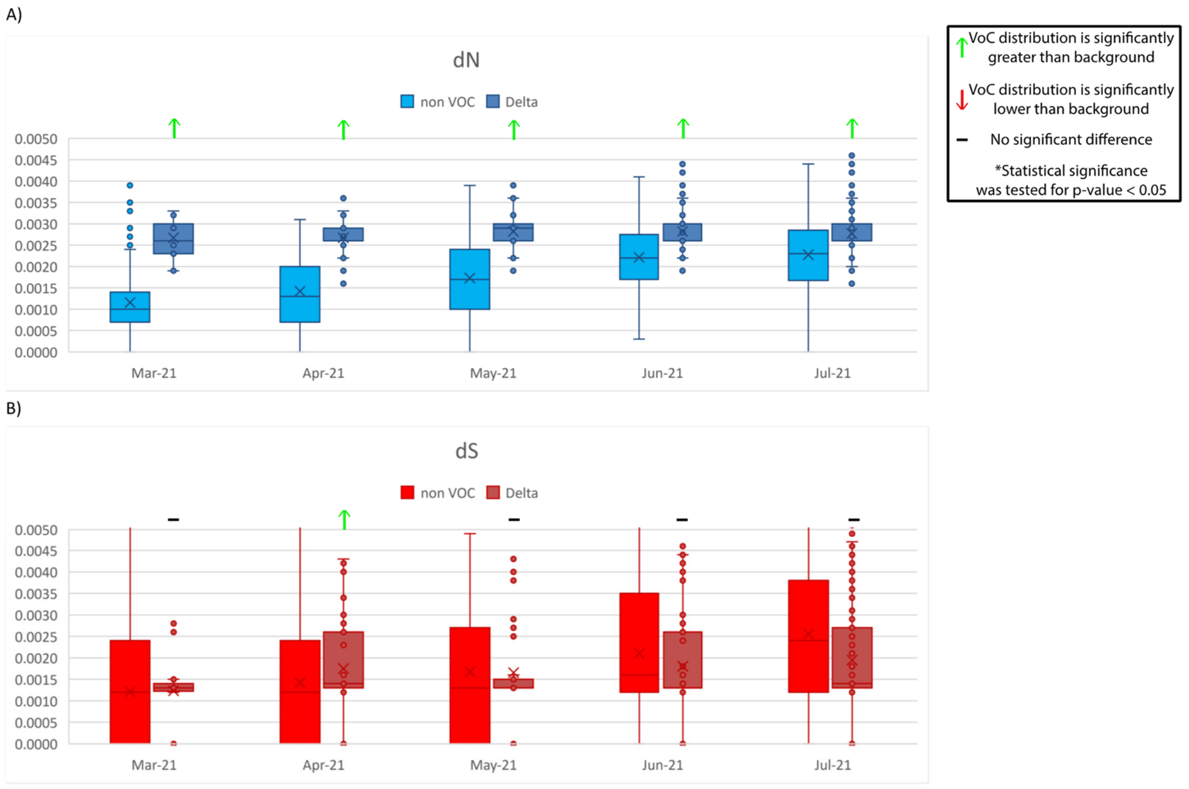

Supplementary Figure S4. Delta lineage Spike. A) Comparison of average dN rates between the Delta lineage and the non-VoC/VoI background lineages, for each month. B) Comparison of average dS rates between the Delta lineage and the non-VoC/VoI background lineages, for each month.

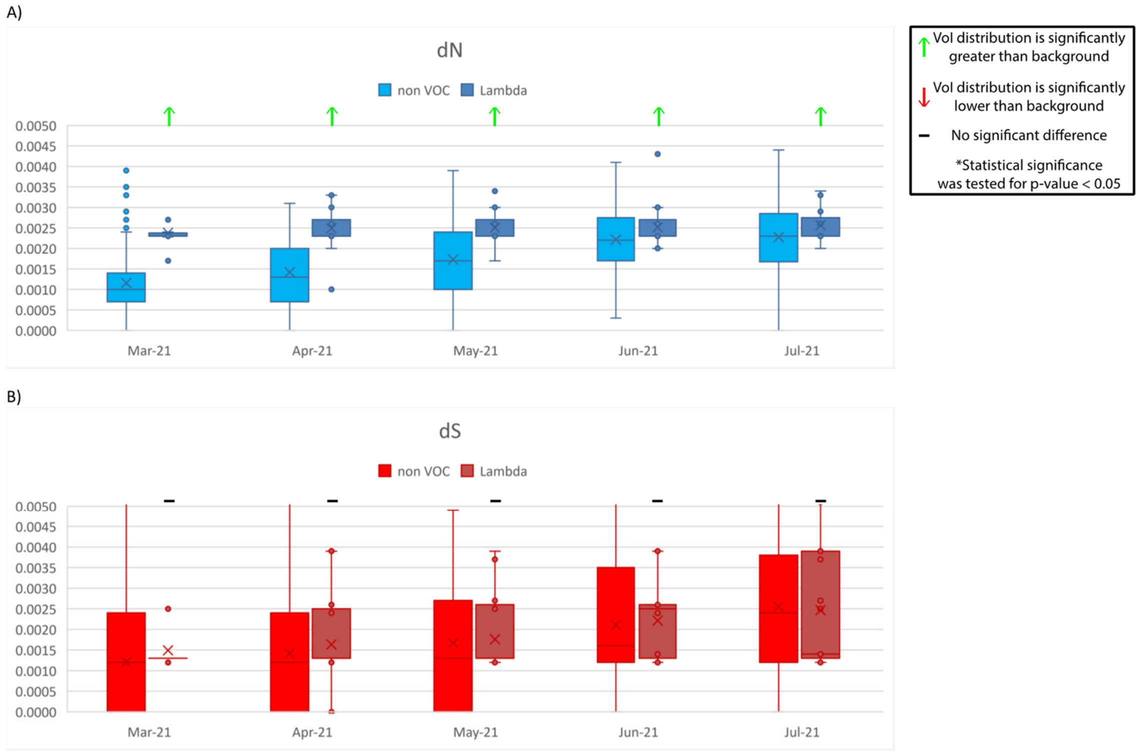

Supplementary Figure S5. Lambda lineage Spike. A) Comparison of average dN rates between the Lambda lineage and the non-VoC/VoI background lineages, for each month. B) Comparison of

average dS rates between the Lambda lineage and the non-VoC/VoI background lineages, for each month.

Conservation of substituted amino acid residues in other Sarbecoviruses.

We investigated whether a specific AAS of a given SARS-CoV-2 variant was not observed in the homologous site of any other Sarbecovirus. Thus, for each ORF/nsp, we aligned (with MAFFT) the Wuhan-Hu-1 sequence, representative sequences from each of the 6 SARS-CoV-2 variant lineages, 78 Sarbecovirus sequences that were analyzed by (Lytras et al., 2021) and 5 Sarbecovirus sequences isolated from Laos (Temmam et al., 2021) that are considered among the closest known relatives of SARS-CoV-2. Multiple alignments were manually inspected and we only retained very well aligned regions and sites that had an AAS in any of the 6 SARS-CoV-2 variant lineages. The aligned sites are shown in Supplementary figure S**6**.

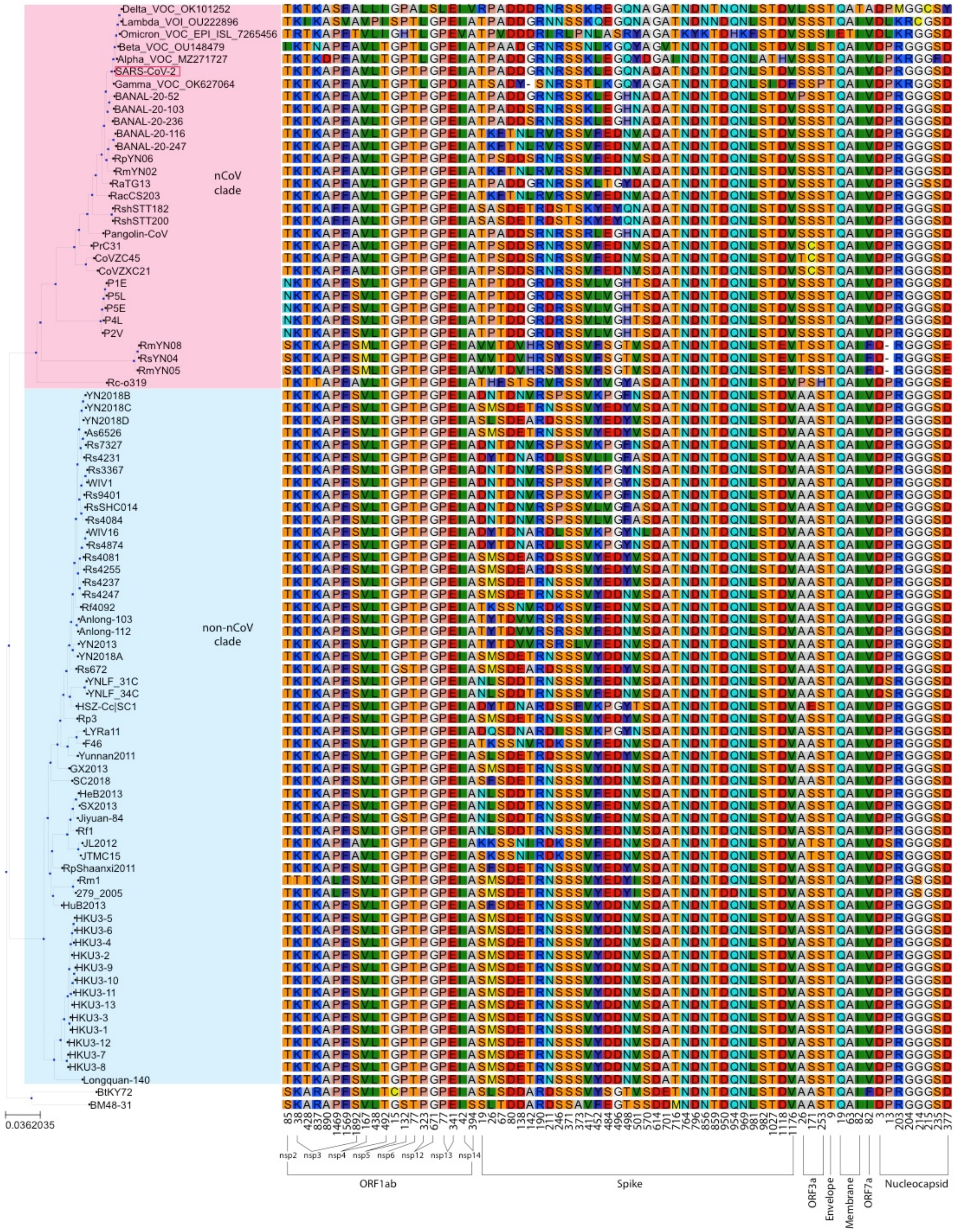

Supplementary figure S6. Sixty nine amino acid substitutions (AAS) of high frequency (≥50) in SARS-CoV-2 lineages (5 VoCs and 1 VoI) that have not been observed in any other Sarbecovirus (outside SARS-CoV-2 lineages). These mutations are found in various ORFs. On the left side is the phylogenetic tree of the sequences, based on ORF1ab. On the right side, each column corresponds to the homologous amino acids, based on well conserved and well aligned regions. At the bottom is the coordinate of each amino acid site in the NCBI reference Wuhan-Hu-1 strain.

The effect of P132H substitution of the Omicron variant in nsp5, the chymotrypsin-like protease (3CL main protease, or M^{pro})

M^{pro} is essential in viral replication as it enables production of the non-structural proteins by cleaving the large polyproteins pp1a and pp1ab. M^{pro} forms a functional dimer mainly through the helical N-terminal domain III (Supplementary Figure S7A), with its active site located at the cleft of domains I (8–101) and II (102–184). The active site cavity is located on the surface of M^{pro} with a catalytic dyad comprising His41 and Cys145 (Supplementary Figure S7B). Cys145 is the target of small-molecule covalent inhibitors of M^{pro}, including the oral drug PF-07321332 developed by Pfizer [1]. PF-07321332 under the brand name Paxlovid is co-administered with a low dose of ritonavir, an HIV protease inhibitor, to enhance its bioavailability. The substitution of Pro by His at position 132 of nsp5 raises the question of whether Paxlovid will be effective against the Omicron variant. Investigation of the ligand-free M^{pro} structures reveals that Pro132 is far from the active site (22–24 Å from the catalytic dyad in PDB ID 6WQF) [2], therefore its substitution can be regarded as insignificant to the catalytic activity of the protease. However, considering that the side-chain of Pro132 is buried to a large extent (~10% relative buried surface area), substitution by His132 in the Omicron variant could result in perturbation of the M^{pro} dynamics, either by influencing the dimeric interface or by affecting other residues that flank the active site cavity.

To scrutinize this assumption, we performed a comparative study of the structural dynamics in the P132 and H132 variants of M^{pro} dimer. In order to obtain statistically meaningful observations, 3 high-resolution structures of inhibitor-free M^{pro} were used as starting points for atomistic molecular dynamics (MD) simulations in explicit solvent (see Computational Methods further below in Supporting Information). The timescale of 200 ns and the parameters used herein are in accordance with recent MD studies of serial femtosecond X-ray crystallographic structures of M^{pro} [3] . Both variants displayed well-converged dynamics within this timescale (Supplementary Figure S**8**) and very similar structural dynamics as displayed by the root-mean-square fluctuations (RMSF) of the M^{pro} backbone (Supplementary Figure S**9**). The per residue difference in their absolute RMSF values displays a minimal perturbation of the dynamics by the substitution of Pro132 by His132 (Supplementary Figure S7C) as evident by the low mean absolute RMSF difference. Even for some residues that display a noticeable RMSF difference, for example in residues 46–52 (Supplementary Table S1), the statistical significance is low given that this region displays the highest flexibility (RMSF values, Supplementary Figure S**9**) in the simulations of M^{pro} dimer. Principal component analysis of the MD simulations suggest that the dominant motions of M^{pro} dimer are also similar in the two variants (Supplementary Figure S**10**). Taken together, our results support the hypothesis that the P132H substitution in M^{pro} of the Omicron variant will have a negligible effect in the catalytic activity and inhibitor binding/efficacy.

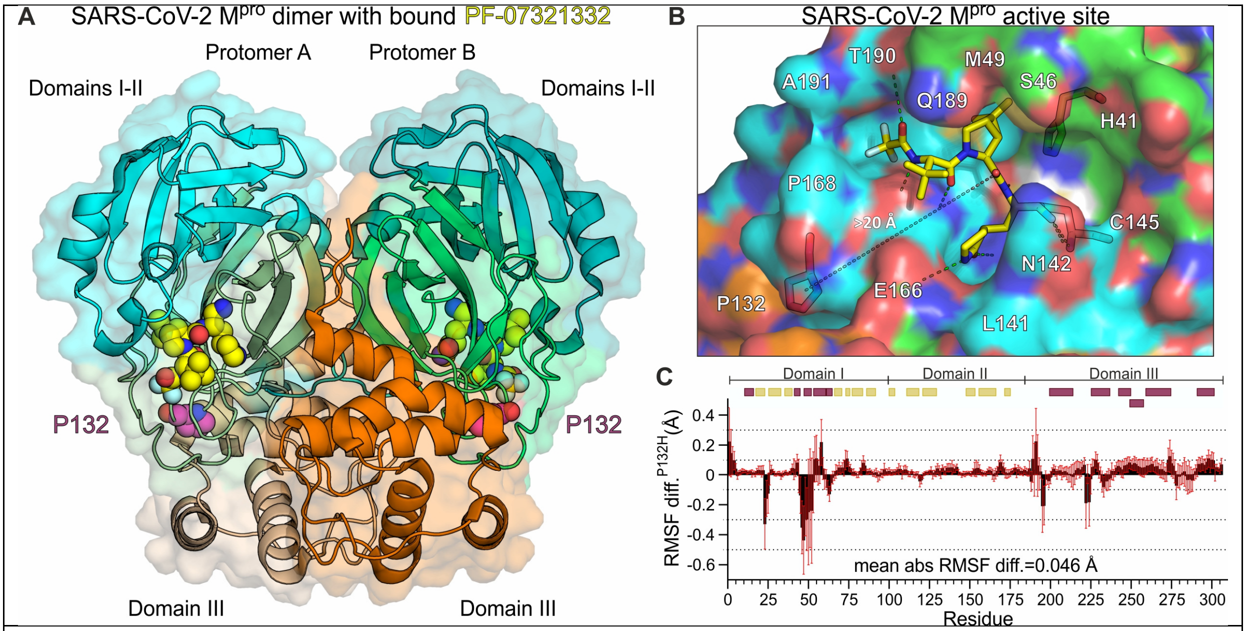

Supplementary Figure S7. (A) Crystallographic structure of SARS-CoV-2 main protease (M^{pro}, nsp5) in complex with the covalent inhibitor PF-07321332, the active ingredient of Paxlovid (Pfizer) [1]. The M^{pro} dimer is color-coded with domain I in green, domain II in cyan and domain III in orange, whereas the inhibitor is shown with spheres colored with yellow C, red O, blue N and light blue F. The variant position at Pro132 is indicated with magenta spheres. (B) Close-up view of the M^{pro} active site in surface representation with the inhibitor bound to Cys145 shown as sticks. Residues from domains I-II that flank the active site are labeled and dashed lines indicate M^{pro}–inhibitor hydrogen bonding interactions. Although Pro132 appears close to the active site, its distance is >20 Å from the active site cavity (22.5 Å from the P_1–P_2 amide bond of the inhibitor). (C) Bar plot of the difference in the mean root-mean-square fluctuations (RMSF) of the backbone C^{α} atoms between the two variants at position 132 (Pro132–His132). Error bars indicate the standard error extracted from 3 MD simulations of each M^{pro} variant, each comprising 2 protomers (N=6 values for each residue). The mean absolute RMSF difference of <0.05 Å over all residues indicates a minor perturbation of the M^{pro} dynamics by the P132H substitution. A few residues that display absolute differences >0.25 Å could have been considered, however, these are of low statistical significance (t-test values >0.1). The upper panel is a graphical representation of the secondary structure assignment of each domain (red: helix, yellow: sheet) taken from PDB ID: 7RFW [1].

Computational Methods

Nsp5/3CL main protease (M^{pro}) structures

To obtain statistically meaningful observations from the molecular dynamics simulations of the M^{pro} dimer, we employed 3 sets of initial coordinates from 3 high-resolution X-ray structures of ligand-free M^{pro}. In particular, we selected the room-temperature (293 K) X-ray structure of unliganded M^{pro} that was resolved at 2.30 Å (PDB ID: 6wqf) [2], the ambient temperature (294 K) structure resolved by serial femtosecond X-ray crystallography at 1.90 Å resolution (PDB ID: 7cwb) [3], and a high-resolution X-ray crystal structure (data collected at 100 K) that was resolved at 1.20 Å resolution (PDB ID: 7k3t). Although the structures are highly similar, we reasoned that initiating the simulations from three different states would allow for better sampling of the conformational space at the submicrosecond timescale. Coordinates of the 3 structures were retrieved from RCSB and the M^{pro} dimer was generated by crystallographic symmetry operators. The resolved water molecules were retained, whereas all other non-protein atoms were discarded.

Protonation states of histidine residues in the M^{pro} dimer were assigned using the H++ server (pH=7.4, ionic strength of 0.15 M and default dielectric constants) [4]. Acidic and basic residues were assigned as negatively or positively charged, respectively, whereas no histidine residue was assigned its protonated form. The initial structures of the H132 variant were obtained by substituting the side-chain C^{γ} and C^{δ} atoms of P132 with an imidazole ring. Re-calculation of the pKa values for the H132 variants with H++ indicated that H132 is probably at its protonated form under physiological conditions, possibly due to its proximity to the acidic D197 and E240. Therefore, H132 was assigned as positively charged in both protomers of the three M^{pro} dimeric structures. The catalytic C145 residue was set to be uncharged (thiol form) in accordance with the crystallographic observations of ligand-free M^{pro} at room temperature [2].

The 6 simulation systems of the two M^{pro} variants were prepared using the XLEaP module of AMBER v20 [5], with force field parameters assigned from the AMBER ff14SB dataset. Truncated octahedral simulation boxes were generated with pre-equilibrated TIP3P solvent molecules so that the minimum distance between the solute and the box edges was at least 10 Å. The required number of Na^+ ions was then added so as to neutralize the systems (8 for P132 and 6 for H132), whereas no other counterion was added.

Molecular dynamics simulations

All simulations were performed using the GPU-accelerated PMEMD module of AMBER [6] on an NVIDIA RTX-equipped workstation. A time step of 2 fs was employed in conjunction with the SHAKE algorithm to constraint hydrogen atoms at their equilibrium distance. The Langevin thermostat with a collision frequency of 1.0 ps^{-1} was used to regulate temperature and the Berendsen weak-coupling algorithm with a relaxation time of 1.0 ps to regulate pressure. The particle mesh Ewald summation method was used to treat long-range electrostatic interactions with a tolerance of 10^{-6} and the real space cut-off was set to 10 Å. The convergence criterion in energy minimizations was set to the energy gradient < 0.01 kcal·mol^{-1}·Å^{-1}, while all other parameters were kept to their default values.

The system equilibration phase of 10 ns was not used in data analysis and comprised the following steps. Energy minimization was initially carried out to relax the solvent molecules followed by a short 100-ps equilibration of the solvent in the isothermal–isobaric (*NPT*) ensemble at T=300 K and P=1 bar, while restraining all non-hydrogen atoms of the M^{pro} dimer. A second energy minimization was then performed with positional restraints on the C^{α} atoms of M^{pro} (force constant of 10 kcal·mol^{-1}·Å^{-2}) before heating the system gradually from 10 to 300 K through 400 ps of simulation under constant volume (*NVT*). The positional restraints were then gradually decreased from 10 to 0.1 kcal·mol^{-1}·Å^{-2} through 3 rounds of 200-ps *NPT* simulations (T=300 K, P=1 bar) in order to equilibrate the density of the systems. After that, 9 ns of unrestraint simulations in the *NPT* ensemble were carried out before each production run of 200 ns that was carried out under the conditions described above. Trajectories were updated every 2,500 steps (5 ps) for a total of 40,000 frames in each 200-ns simulation. An aggregate of 600 ns of MDs were obtained for each variant system for processing.

Trajectory analysis

Analysis of the trajectories was carried out using the CCPTRAJ module [7] of AMBER after root-mean-square (RMS) fitting of all protein C^{α} atoms. Principal component analysis was carried out with CCPTRAJ using only the C^{α} atoms of all trajectory frames for each M^{pro} variant. Statistical analysis was performed with R-based scripts and plots were generated using Grace. The energy landscapes (PMF) were obtained from Boltzmann-weighted projection of the trajectories along the first two principle components (PC-1 and PC-2) of each system. Porcupine plots to illustrate the protein

backbone motions along PC-1 and PC-2 were generated using the NMWiz extension of VMD v1.9 [8]. All other figures were generated using the open-source build of PyMOL v2.3.

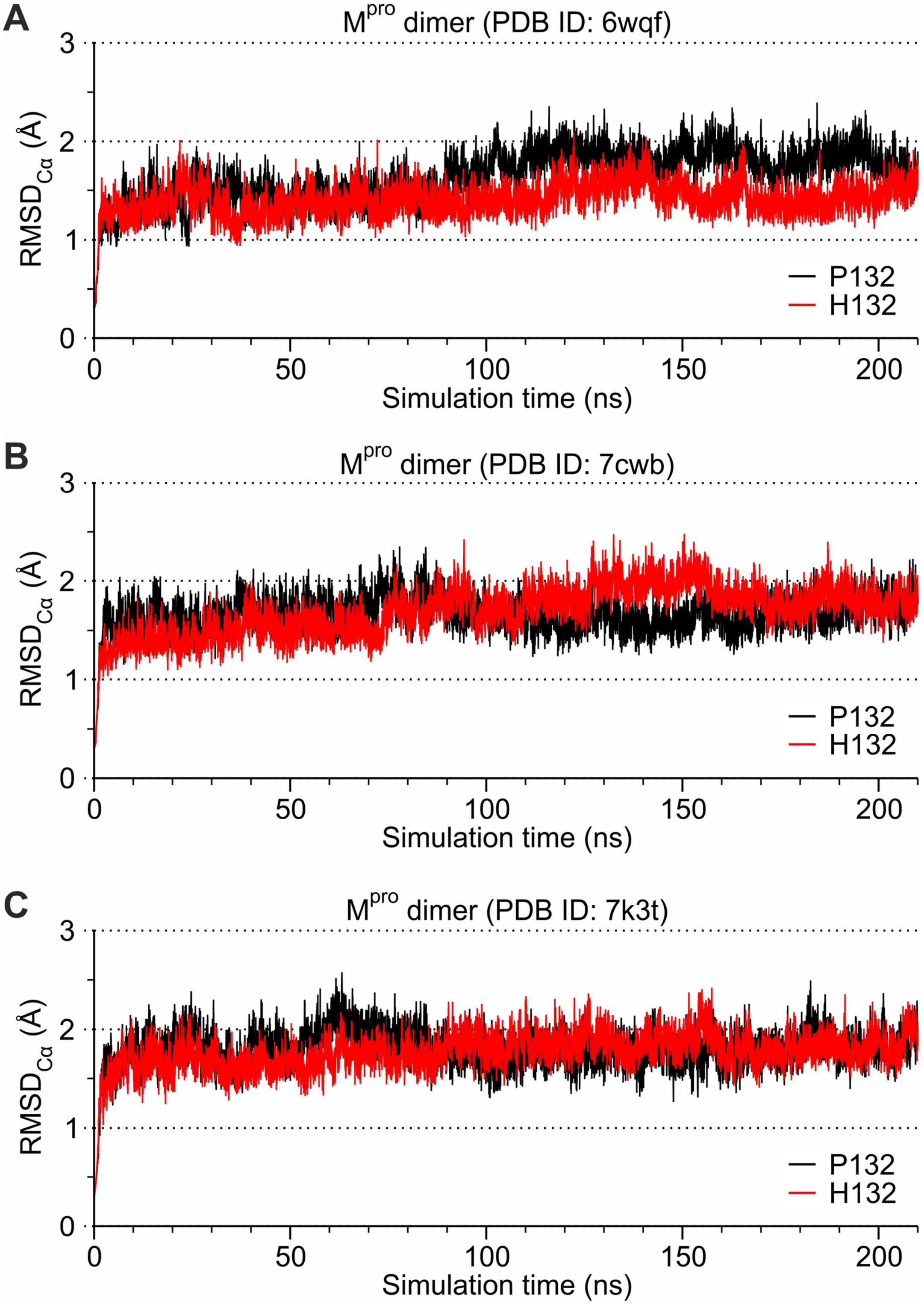

Supplementary Figure S8. Plots of the root-mean-square deviation (RMSD) of all Cα atoms of the M^{pro} dimer as a function of simulation time. Data were extracted from MD simulations starting from 3 experimental structures with PDB IDs: 6wqf (A), 7cwb (B) and 7k3t (c). Black lines are for the Wuhan-hu-1 nsp5 protease (P132) and red lines for the Omicron variant (H132). The initial 10 ns of equilibration were not used in the analysis of trajectories.

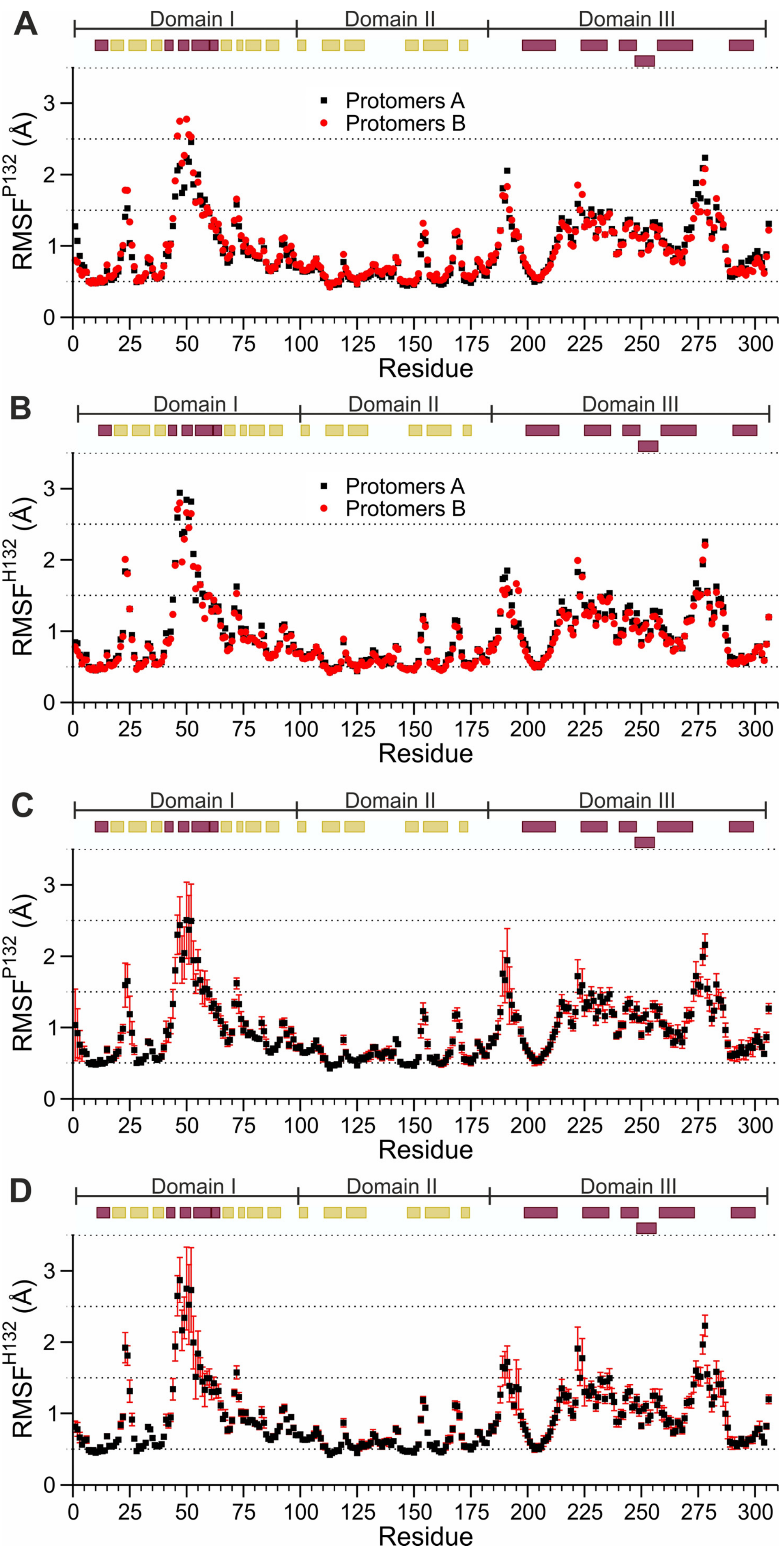
A
Domain I
Domain II
Domain III
Protomers A
Protomers B
RMSF^P132 (Å)
Residue
B
Domain I
Domain II
Domain III
Protomers A
Protomers B
RMSF^H132 (Å)
Residue
C
Domain I
Domain II
Domain III
RMSF^P132 (Å)
Residue
D
Domain I
Domain II
Domain III
RMSF^H132 (Å)
Residue

Supplementary Figure S9. Plots of the atomic root-mean-square fluctuations (RMSF) of the M^{pro} Cα atoms. Mean RMSF values per residue of each protomer were calculated from 3 MD simulations of the P132 variant (A) and 3 MD simulations of the H132 variant (B). Mean RMSF values per residue of both M^{pro} protomers calculated from the simulations of P132 (C) and H132 (D) M^{pro}. Error bars indicate the standard deviation of each point from N=6 values (3 simulations x 2 protomers).

Supplementary Table S1. Difference of the mean RMSF values between the M^{pro} Cα atoms of the two variants (P132–H132) obtained from the 200 ns MD simulations (Figures S2C,D). The RMSF$_{diff}$ and standard error (SE for N=6) are given in Å, whereas residues in bold indicate active site and flanking residues of importance to substrate or inhibitor binding.

ResID	RMSF$_{diff}$	SE
1	0.22	0.23
2	0.14	0.16
3	0.10	0.09
4	0.10	0.06
5	0.04	0.04
6	-0.01	0.02
7	0.01	0.02
8	0.02	0.02
9	0.03	0.02
10	0.03	0.01
11	0.02	0.01
12	0.02	0.01
13	0.02	0.01
14	0.02	0.01
15	0.01	0.03
16	0.01	0.02
17	0.00	0.02
18	0.02	0.02
19	0.03	0.03
20	0.03	0.03
21	0.02	0.04
22	0.03	0.03
23	-0.33	0.17
24	-0.16	0.12
25	-0.12	0.13
26	0.01	0.08
27	0.04	0.03
28	0.03	0.01
29	0.01	0.02
30	0.00	0.01
31	0.01	0.01
32	0.01	0.02
33	-0.01	0.02
34	0.01	0.02
35	0.01	0.02
36	0.02	0.01
37	0.00	0.01
38	0.01	0.01
39	0.02	0.02
**40**	**0.05**	**0.03**
**41**	**0.04**	**0.07**
42	0.04	0.05
43	0.08	0.06
44	-0.01	0.11
45	-0.14	0.12
**46**	**-0.35**	**0.18**
47	-0.44	0.23
48	-0.21	0.20
49	-0.30	0.21
**50**	**-0.25**	**0.35**
51	-0.16	0.33
52	-0.24	0.35
53	-0.05	0.20
54	0.10	0.15
55	0.11	0.16
56	0.02	0.09
57	0.06	0.12
58	0.22	0.15
59	0.04	0.07
60	-0.03	0.07
61	-0.02	0.06
62	-0.09	0.05
63	-0.13	0.05
64	-0.05	0.05
65	-0.02	0.04
66	-0.01	0.03
67	0.02	0.05
68	0.03	0.03
69	0.05	0.04
70	0.03	0.03
71	0.05	0.03
72	0.04	0.05
73	0.10	0.06
74	0.11	0.05
75	0.06	0.03
76	0.04	0.03
77	0.02	0.02
78	0.02	0.02
79	0.01	0.02
80	0.00	0.02
81	0.00	0.02
82	0.02	0.02
83	0.05	0.05
84	0.08	0.05
85	0.06	0.03
86	0.02	0.02
87	0.02	0.02
88	0.03	0.02
89	0.02	0.02
90	0.00	0.02
91	0.01	0.02
92	0.01	0.03
93	-0.01	0.04
94	-0.01	0.03
95	0.01	0.03
96	0.01	0.03

ResID	RMSF$_{diff}$	SE
97	0.04	0.04
98	0.02	0.02
99	0.02	0.02
100	0.02	0.02
101	0.02	0.02
102	0.02	0.02
103	0.02	0.02
104	0.02	0.02
105	0.03	0.02
106	0.04	0.04
107	0.04	0.02
108	0.00	0.03
109	0.03	0.03
110	0.04	0.03
111	0.03	0.03
112	0.01	0.02
113	0.00	0.02
114	0.02	0.01
115	0.00	0.02
116	0.00	0.02
117	0.01	0.01
118	0.00	0.02
119	-0.04	0.04
120	-0.04	0.03
121	-0.01	0.02
122	0.01	0.01
123	0.02	0.01
124	0.01	0.01
125	0.02	0.01
126	0.03	0.03
127	0.00	0.03
128	0.01	0.02
129	0.02	0.04
130	0.04	0.03
131	0.03	0.03
132	*0.01*	*0.03*
133	0.04	0.02
134	0.03	0.03
135	0.03	0.03
136	0.05	0.03
137	0.05	0.03
138	0.05	0.03
139	0.05	0.05
140	0.04	0.03
141	0.04	0.02
**142**	**0.05**	**0.02**

ResID	RMSF$_{diff}$	SE
143	0.02	0.02
144	0.01	0.01
**145**	**0.00**	**0.01**
146	0.01	0.02
147	0.00	0.02
148	0.01	0.01
149	0.01	0.01
150	0.01	0.01
151	0.01	0.02
152	0.02	0.02
153	0.04	0.05
154	0.04	0.06
155	0.05	0.04
156	0.04	0.03
157	0.02	0.02
158	0.01	0.02
159	0.01	0.01
160	0.01	0.02
161	0.02	0.02
162	0.03	0.02
163	0.03	0.02
**164**	**0.00**	**0.04**
**165**	**-0.01**	**0.04**
**166**	**0.01**	**0.04**
167	0.02	0.03
**168**	**0.05**	**0.04**
169	0.08	0.05
170	0.06	0.04
171	0.04	0.04
172	0.01	0.04
173	0.01	0.03
174	0.01	0.03
175	0.01	0.02
176	0.02	0.02
177	0.03	0.02
178	0.05	0.02
179	0.02	0.04
180	0.04	0.03
181	0.03	0.03
182	0.02	0.02
183	0.02	0.02
184	0.03	0.02
185	0.02	0.03
186	0.01	0.02
187	0.04	0.05
188	-0.04	0.07

ResID	RMSF$_{diff}$	SE
**189**	**0.11**	**0.16**
**190**	**0.03**	**0.18**
**191**	**0.22**	**0.22**
192	0.06	0.21
193	0.01	0.11
194	0.02	0.04
195	-0.21	0.18
196	-0.21	0.13
197	-0.02	0.05
198	-0.03	0.03
199	-0.04	0.04
200	-0.02	0.04
201	0.02	0.03
202	0.04	0.03
203	0.02	0.03
204	0.00	0.03
205	0.03	0.03
206	0.03	0.03
207	0.01	0.05
208	0.02	0.03
209	0.02	0.03
210	0.01	0.05
211	0.00	0.05
212	0.00	0.05
213	-0.01	0.08
214	-0.01	0.09
215	0.01	0.07
216	0.03	0.06
217	0.04	0.05
218	0.03	0.06
219	0.04	0.05
220	0.05	0.04
221	0.07	0.05
222	-0.19	0.17
223	0.01	0.06
224	-0.18	0.16
225	-0.03	0.10
226	0.05	0.06
227	0.06	0.05
228	0.10	0.05
229	0.08	0.06
230	0.03	0.05
231	0.00	0.05
232	-0.04	0.07
233	-0.08	0.07
234	-0.04	0.05

ResID	RMSF$_{diff}$	SE
235	-0.04	0.07
236	-0.03	0.07
237	-0.02	0.06
238	0.02	0.06
239	0.00	0.04
240	0.02	0.04
241	0.05	0.04
242	0.06	0.04
243	0.05	0.05
244	0.04	0.05
245	0.03	0.06
246	0.06	0.05
247	0.06	0.05
248	0.07	0.05
249	0.08	0.04
250	0.07	0.03
251	0.07	0.04
252	0.08	0.05
253	0.07	0.04
254	0.06	0.04
255	0.05	0.05
256	0.05	0.06
257	0.05	0.06
258	0.05	0.07

ResID	RMSF$_{diff}$	SE
259	0.05	0.05
260	0.06	0.05
261	0.06	0.04
262	0.07	0.05
263	0.07	0.05
264	0.05	0.03
265	0.05	0.04
266	0.06	0.05
267	0.04	0.04
268	0.03	0.05
269	0.04	0.06
270	0.03	0.06
271	0.00	0.05
272	0.02	0.06
273	0.10	0.08
274	0.12	0.11
275	0.08	0.09
276	0.06	0.09
277	0.02	0.08
278	-0.07	0.10
279	-0.01	0.08
280	-0.01	0.08
281	0.00	0.06
282	0.01	0.07

ResID	RMSF$_{diff}$	SE
283	0.02	0.10
284	-0.01	0.10
285	-0.03	0.10
286	-0.01	0.11
287	-0.03	0.08
288	-0.04	0.07
289	0.00	0.04
290	0.02	0.05
291	0.04	0.06
292	0.07	0.05
293	0.07	0.05
294	0.08	0.05
295	0.07	0.04
296	0.06	0.05
297	0.09	0.05
298	0.11	0.06
299	0.07	0.06
300	0.10	0.05
301	0.10	0.05
302	0.01	0.06
303	0.05	0.02
304	0.04	0.01
305	0.04	0.04
306	0.07	0.04

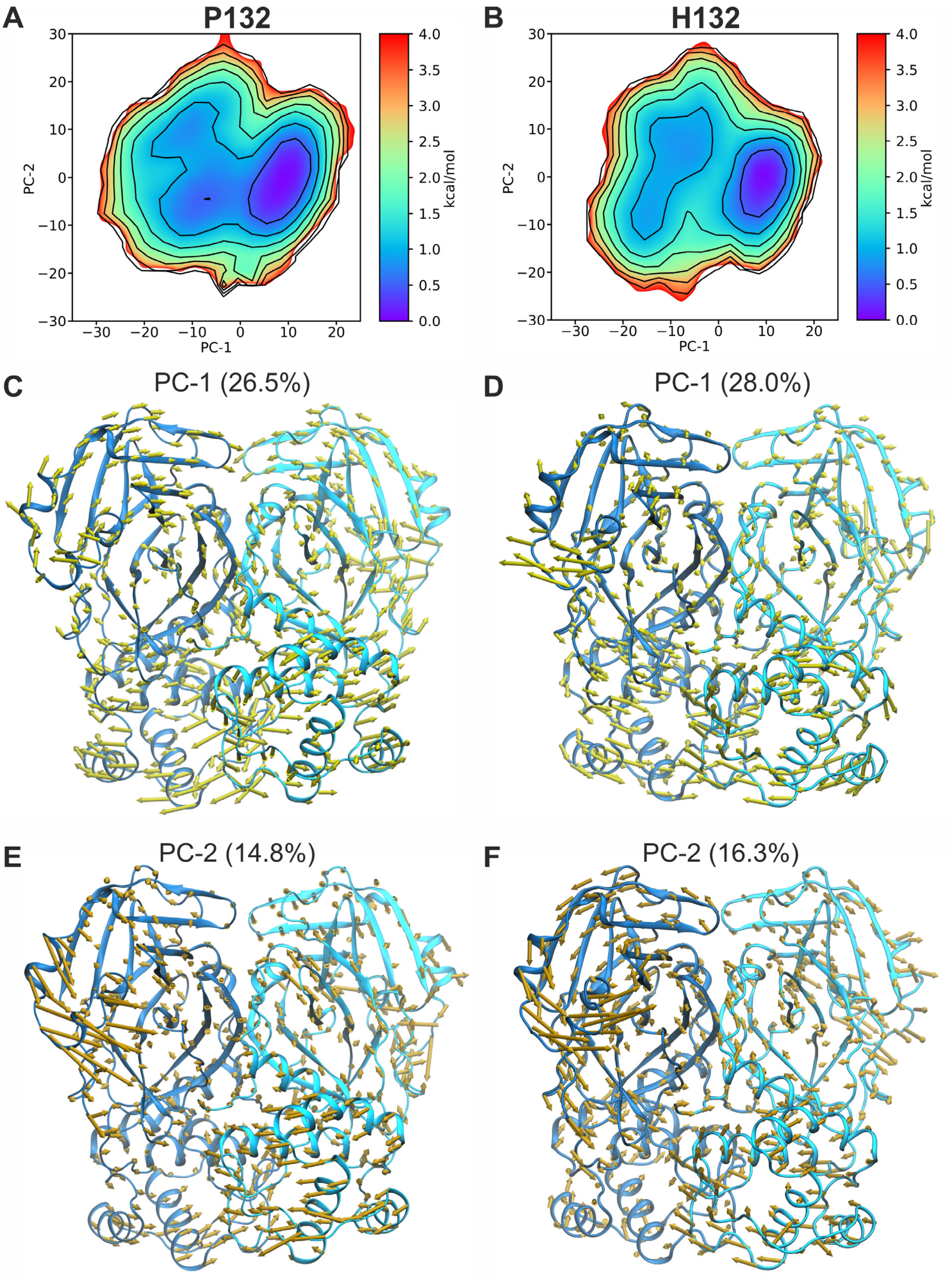

Supplementary Figure S10. Free-energy landscapes of the P132 (A) and the H132 (B) M^{pro} dimer, which were estimated from projection of the simulated trajectories onto the subspace of the first two principal components (PC-1, PC-2). The contribution of each eigenvector to the total motion is given as percent in the parentheses. (C–F) Porcupine plots illustrating the backbone motions along PC-1 and PC-2 for P132 (C, E) and H132 (D, F) M^{pro} dimer. Protomers A are blue and protomers B are cyan.

Nucleocapsid AAS are mostly located at intrinsically disordered regions.

Apart from the Spike, the Alpha, Gamma and Lambda variant lineages had a statistically significant over-representation of AAS for the Nucleocapsid as well. A similar trend (for Nucleocapsid) was observed by [9]. Closer inspection (see Supplementary Figure S**11**) revealed that most of these mutations are located at the N-terminal, the linker and the C-terminal regions of the protein, that are intrinsically disordered [10]. Such disordered regions are known to evolve fast and may be removed from multiple alignments when including more divergent sequences/taxa. Very few AAS were found at the structured and highly conserved RNA-binding and dimerization domains (see Supplementary Figure S**11**).

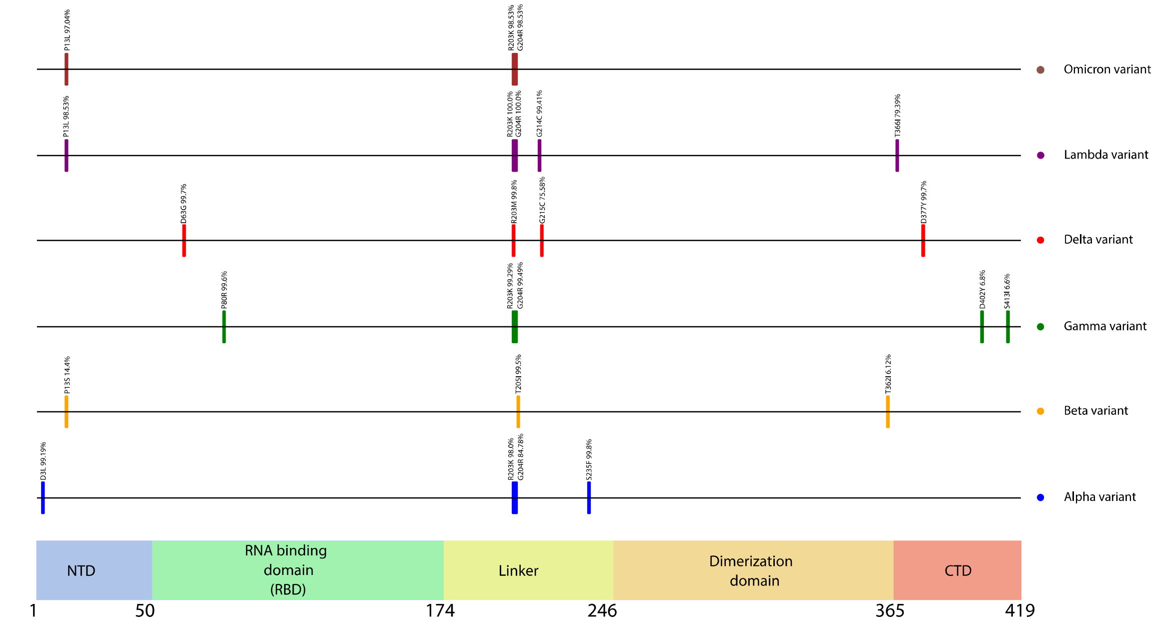

Supplementary Figure S11. Amino acid substitutions (AAS) of the selected variant lineages (compared to Wuhan-Hu-1), across the Nucleocapsid. The N-terminal domain (NTD), the linker region and the C-terminal domain (CTD) are intrinsically disordered. The observed frequency of each AAS for that lineage is also displayed, above the corresponding vertical bar. Coordinates of the various regions are obtained from the NCBI reference Wuhan-Hu-1 sequence.

Amino acid substitutions situated at the accessory ORFs of the Omicron lineage are less than expected.

Intriguingly, we did not observe any AAS (that passed our frequence filters of ≥5%) located within the accessory ORFs of the Omicron lineage. This under-representation is statistically significant (Hypergeometric test: $p<0.033$), whether we analyzed all AAS (≥5%) or the HF-AAS (≥50%). The Beta and Delta lineages had a statistically significant over-representation or not, depending on the subset of AAS analyzed (≥5% or ≥50%), whereas the Alpha, Gamma and Lambda lineages did not display any statistically significant over/under-representation irrespective of the AAS dataset (≥5% or ≥50%) analyzed. Of note, the accessory ORF9b was excluded from our analyses, because it is embedded within the Nucleocapsid core ORF. When we repeated this analysis by excluding the highly mutated Spike ORF from the other core ORFs, the observed Omicron accessory ORF under-representation was no longer statistically significant, probably due to the overall low number of mutations.

As an extra validation step, we manually inspected the amino acid substitutions of the 21K Omicron lineage in the Nextstrain/GISAID webserver and how they were distributed in the phylogenetic tree (of this lineage). At the time of this analysis (January 5, 2022), 142 sequences from the 21K clade were available, with 5, 1, 3, 2, 4 AAS at ORF3a, ORF6, ORF7a, ORF7b, ORF8 respectively. However, all these AAS were of very low frequency and derived states that emerged later (not present at the common ancestor of the available 21K lineage sequences).

Recurrence of high frequency AAS in more than one VoC lineages

We investigated how many of the 109 HF-AAS (≥50% for a certain lineage) observed in this study were shared by two or more of the six variant lineages, and if they were recurrent.

In our analysis, fifteen of the 109 HF-AAS were shared by two or more of the six variant lineages (see Supplementary Figure S**12** - Supplementary Figure S26). In order to determine if such events were recurrent (homoplasy) mutations, or inherited from a common ancestor (of the six lineages), we investigated the distribution of each of these mutations in the Nextstrain phylogenetic tree that was constructed from more than 3,400 representative sequences from various clades/lineages. Ten of the fifteen mutations were homoplasy events, with two of them at ORF1ab (nsp3 and nsp4), seven of them at the Spike ORF and one at the Nucleocapsid. Two of the five inherited mutations (nsp12:P323L - Supplementary Figure S14; Spike:D614G - Supplementary Figure S21) were present in all 6 lineages. Another two inherited mutations (Nucleocapsid:R203K - Supplementary Figure S25; Nucleocapsid:G204R - Supplementary Figure S26) were present at the common ancestor of the Alpha, Gamma, Lambda and Omicron lineages.

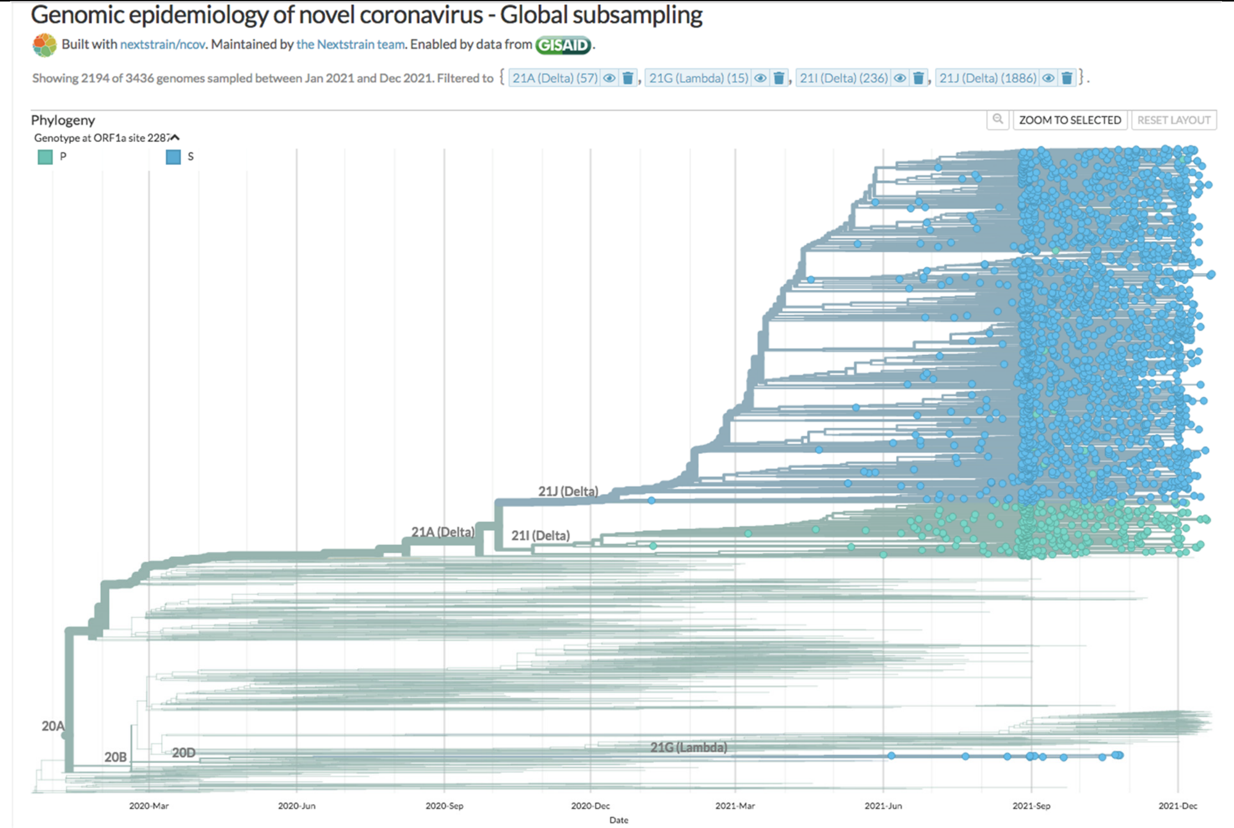

Supplementary Figure S12. Phylogenetic distribution of nsp3_P1469S recurrent mutation, observed in

both Delta and Lambda lineages.

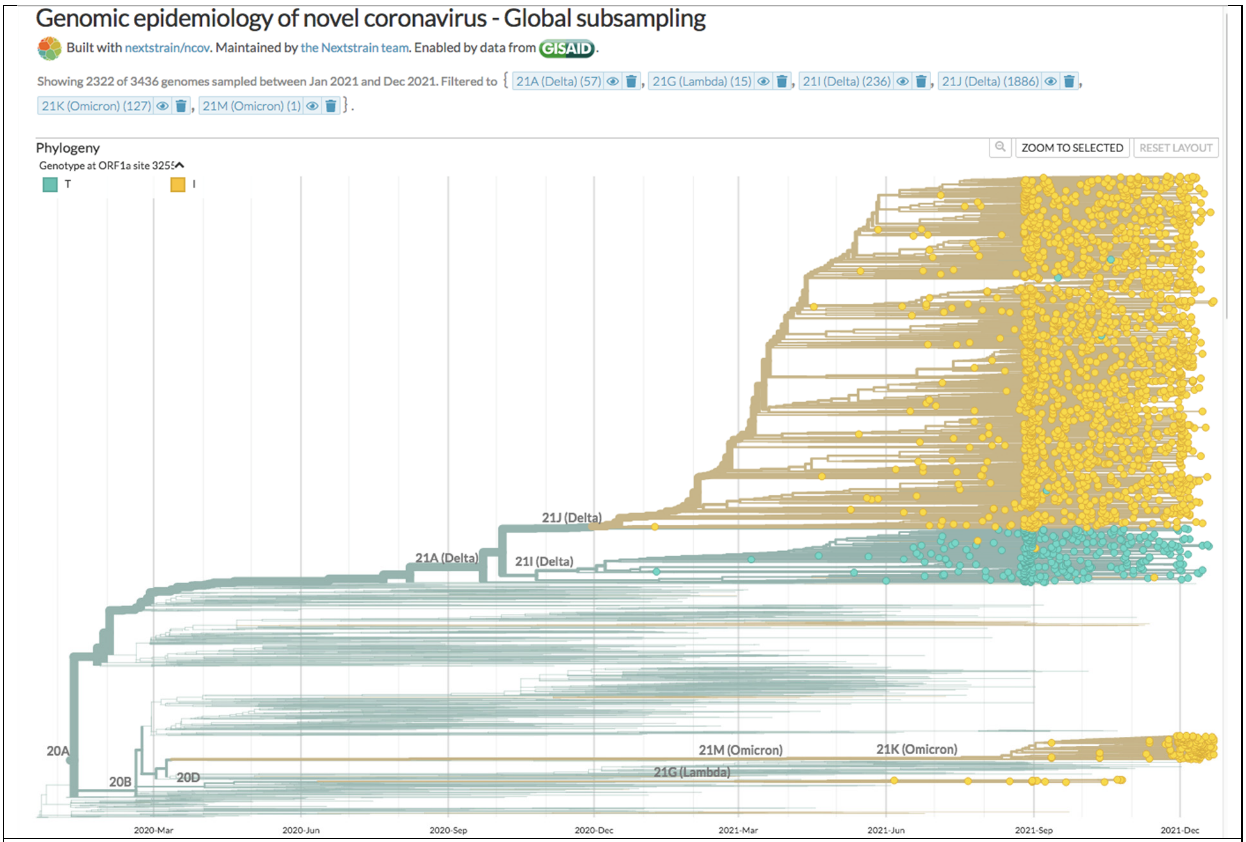

Supplementary Figure S13. Phylogenetic distribution of nsp4_T492I recurrent mutation, observed in Delta, Lambda and Omicron lineages.

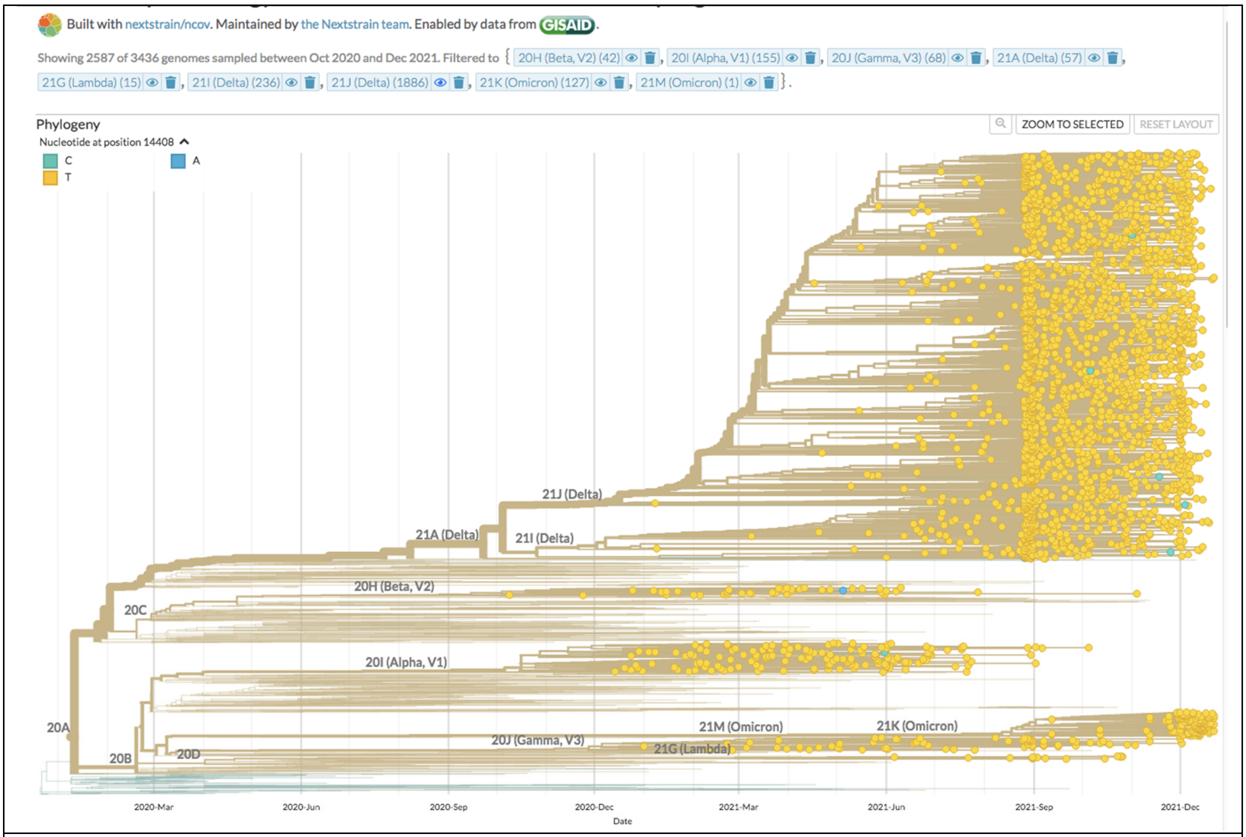

Supplementary Figure S14. Phylogenetic distribution of nsp12_P323L inherited mutation, observed in Alpha, Beta, Gamma, Delta, Lambda and Omicron lineages.

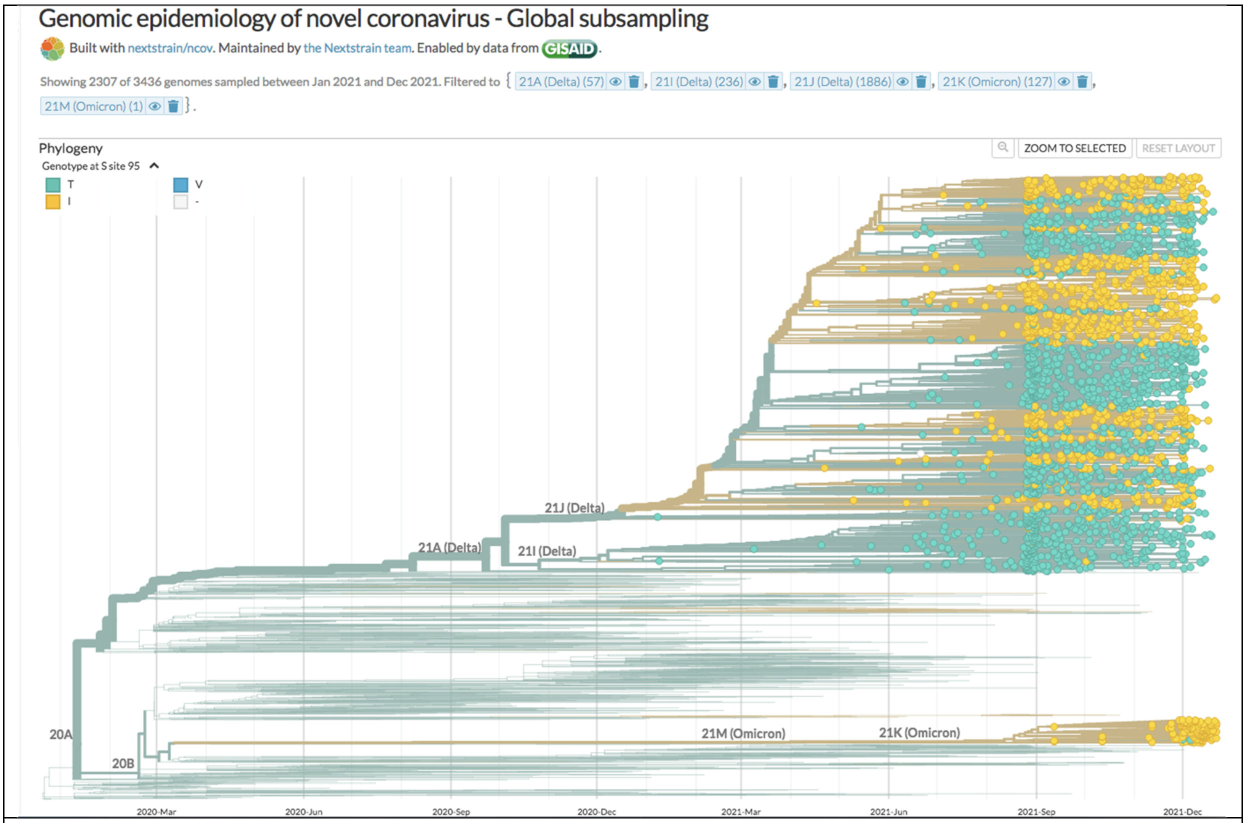

Supplementary Figure S15. Phylogenetic distribution of Spike_T95I recurrent mutation, observed in

Delta and Omicron lineages.

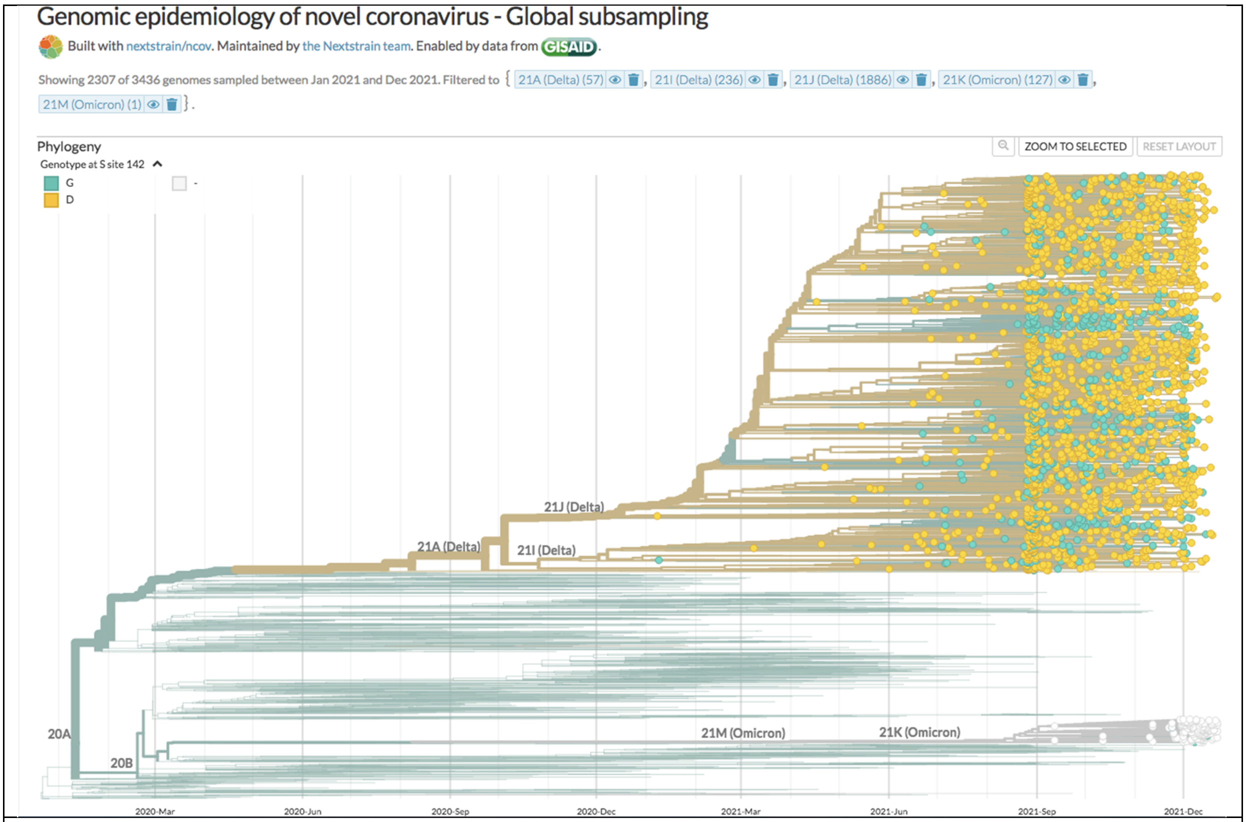

Supplementary Figure S16. Phylogenetic distribution of Spike_G142D recurrent mutation, observed in Delta and Omicron lineages. The mutation of G142D in Omicron is due to nucleotide deletions that change the amino acid.

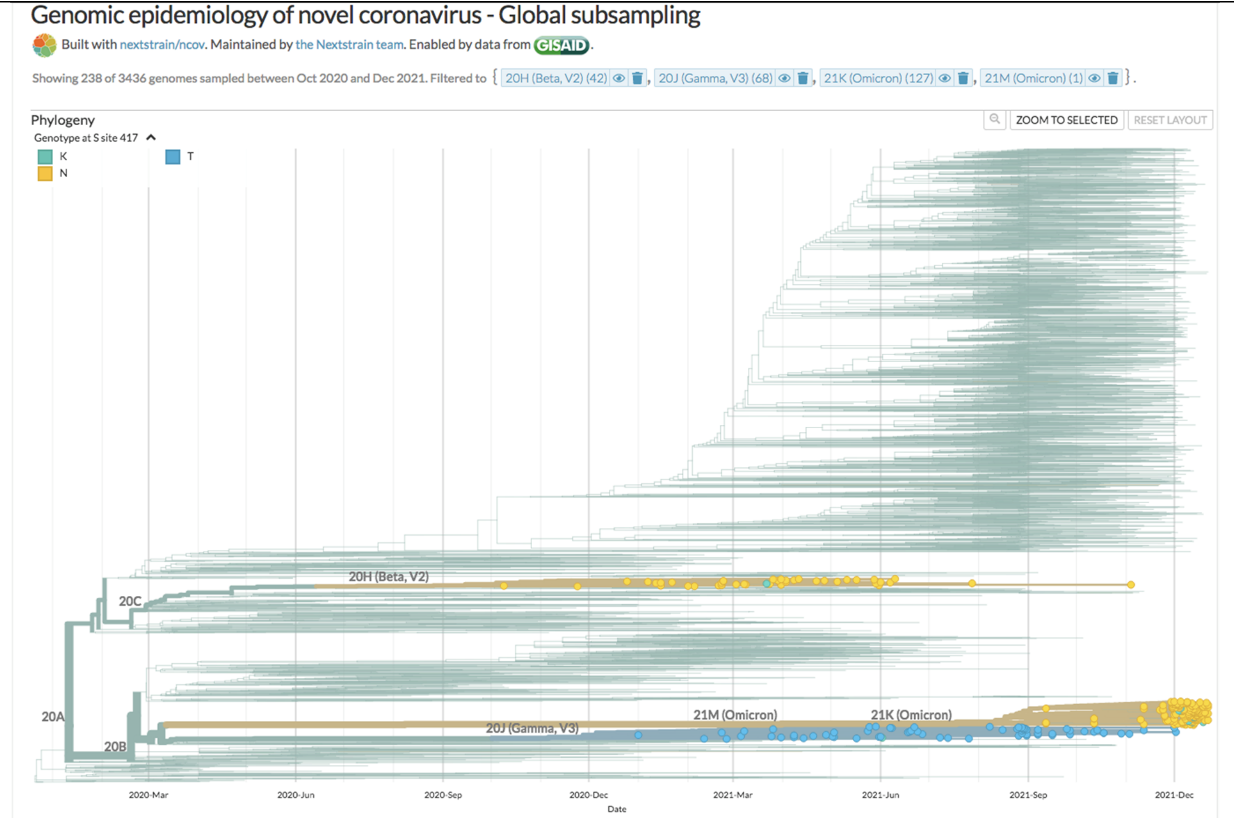

Supplementary Figure S17. Phylogenetic distribution of Spike K417N recurrent mutation, observed in Beta and Omicron lineages.

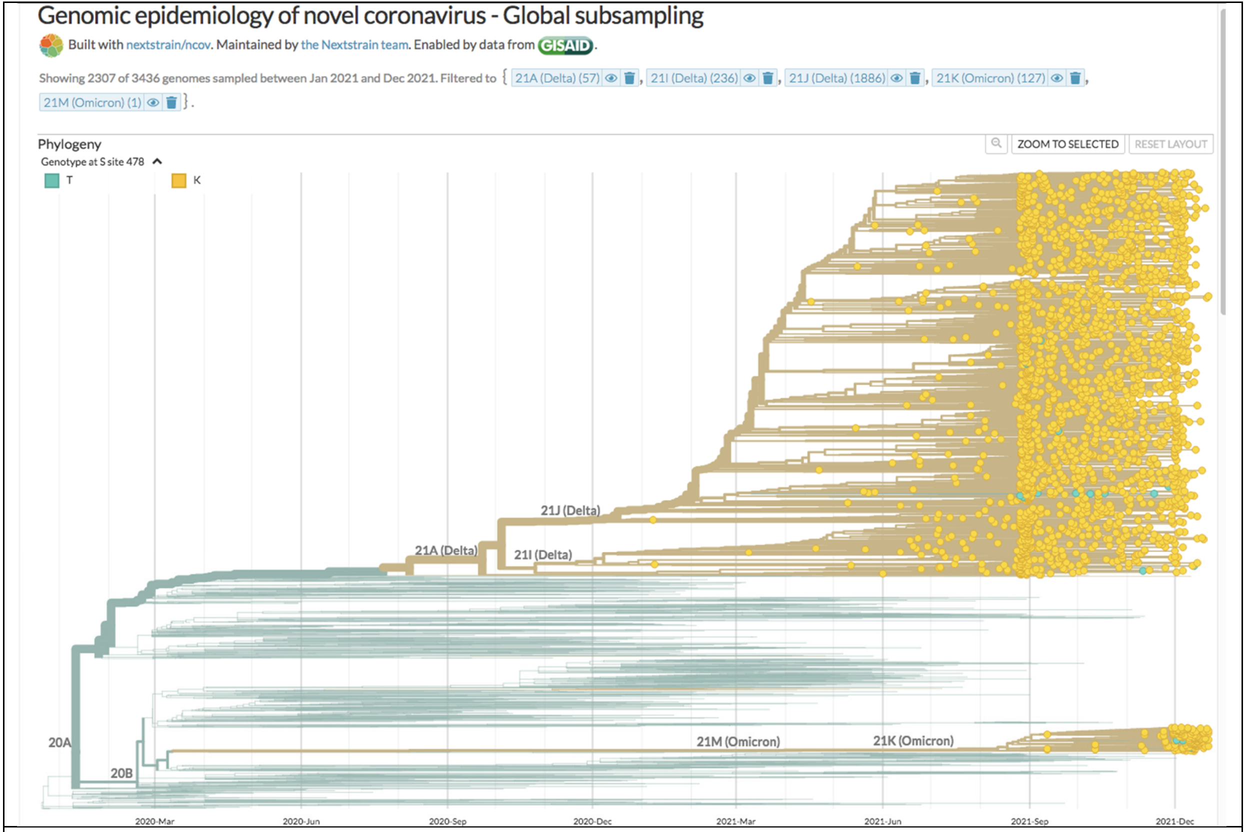

Supplementary Figure S18. Phylogenetic distribution of Spike T478K recurrent mutation, observed in

Delta and Omicron lineages.

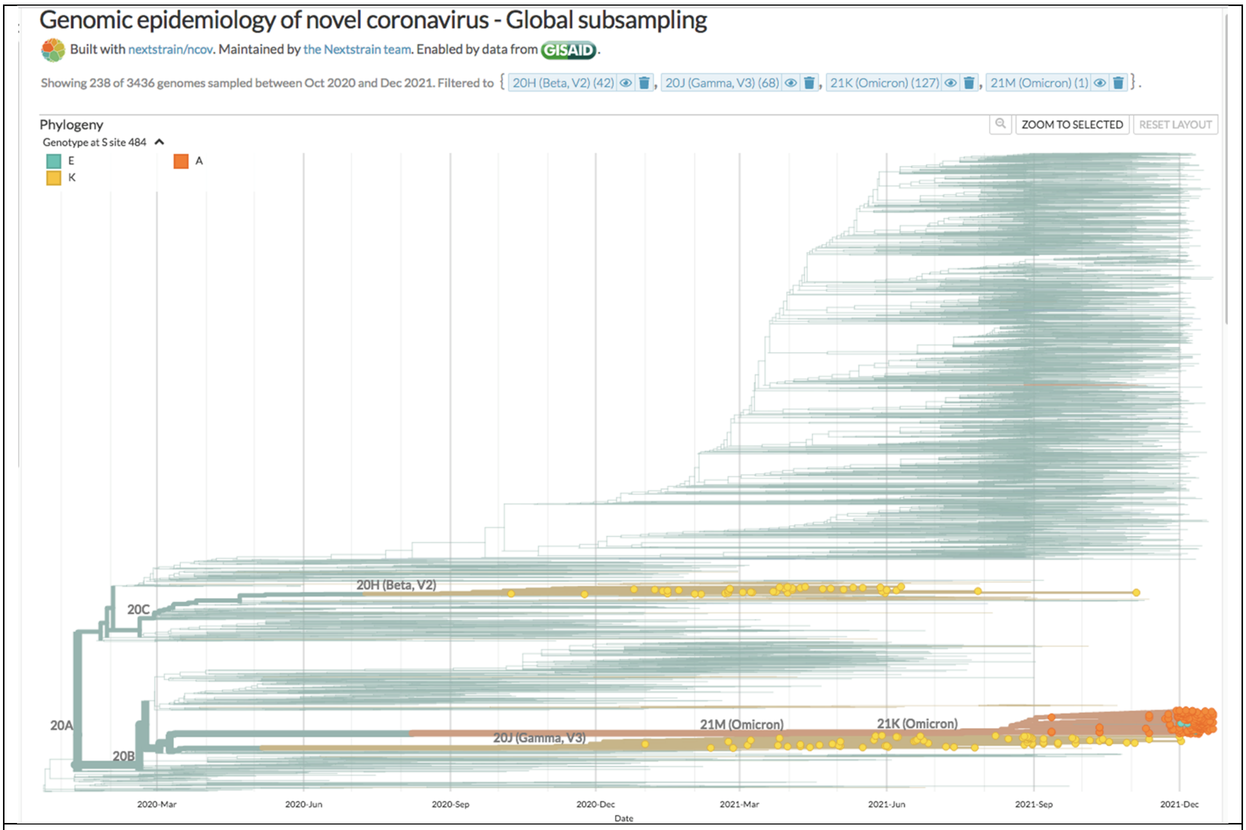

Supplementary Figure S19. Phylogenetic distribution of Spike E484K recurrent mutation, observed in Beta and Gamma lineages.

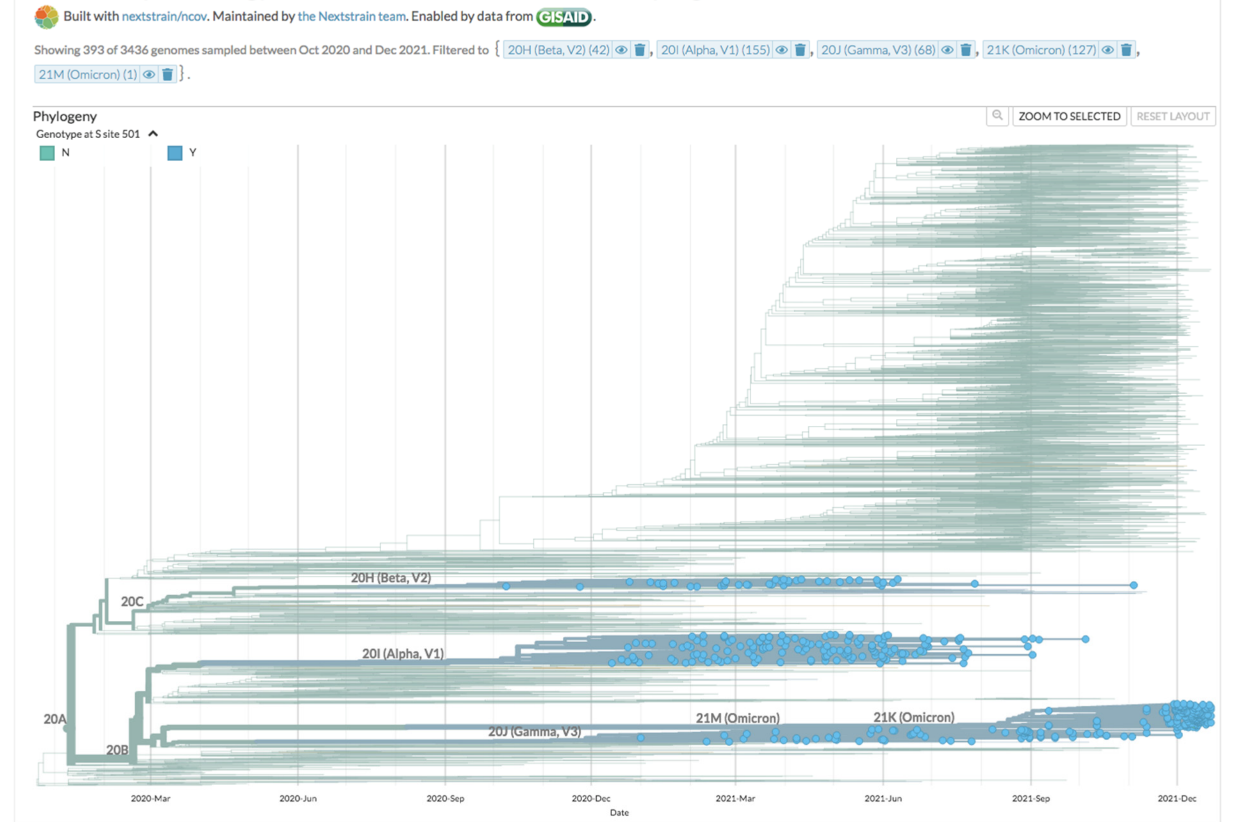

Supplementary Figure S20. Phylogenetic distribution of Spike N501Y recurrent mutation, observed in Alpha, Beta, Gamma and Omicron lineages.

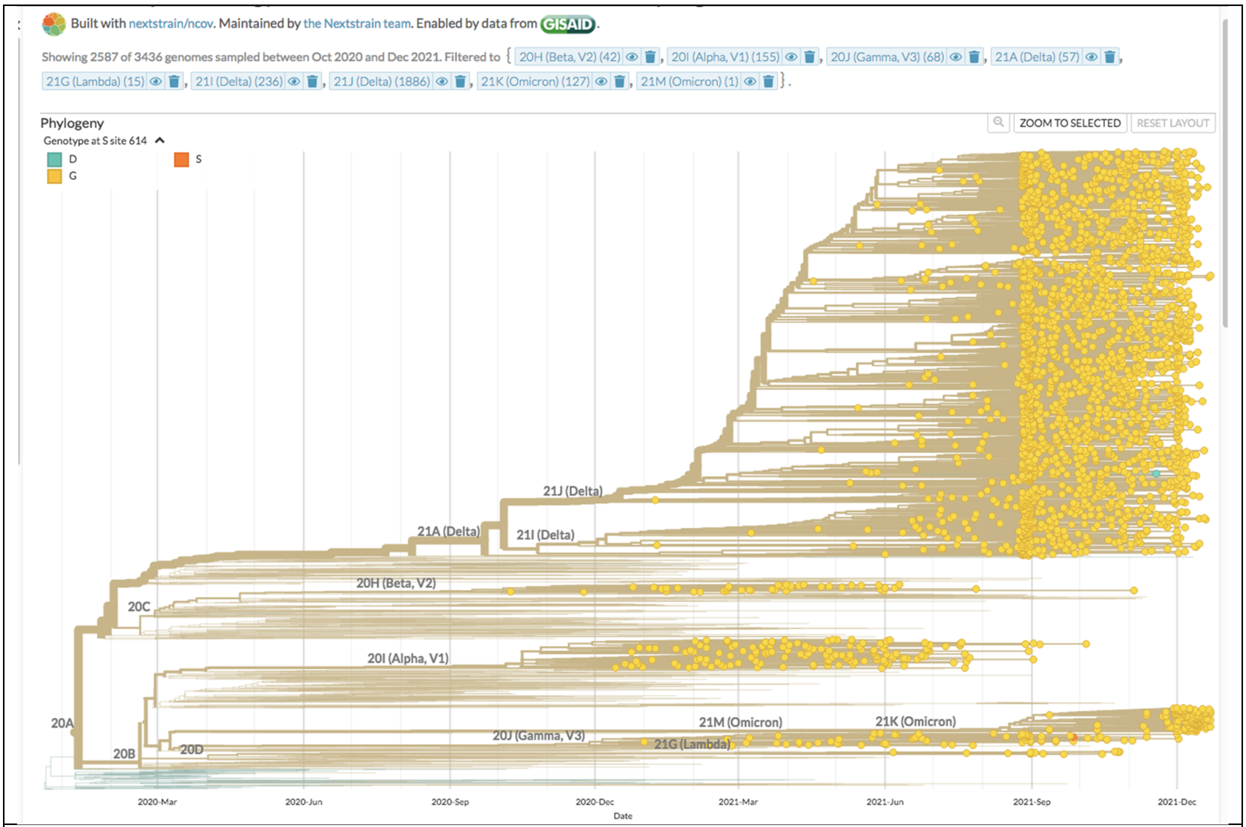

Supplementary Figure S21. Phylogenetic distribution of Spike D614G inherited mutation, observed in

Alpha, Beta, Gamma, Delta, Lambda and Omicron lineages.

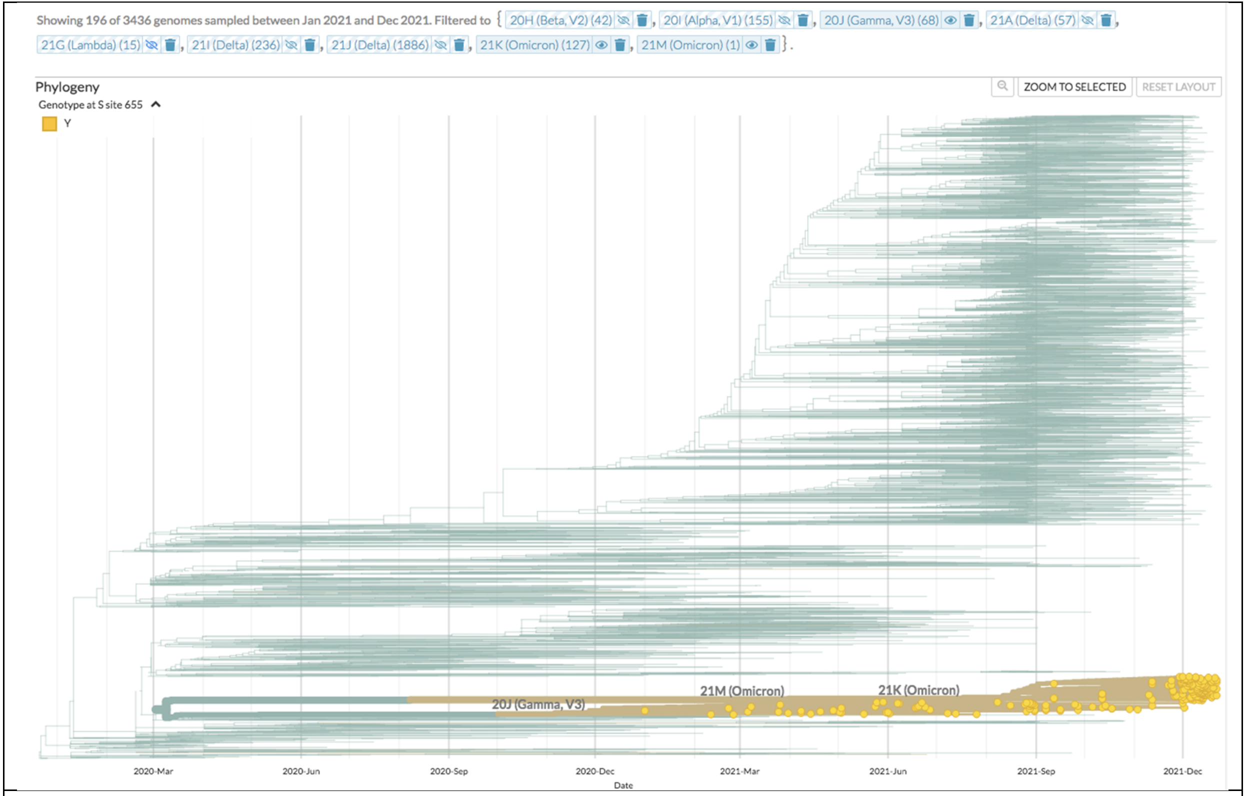

Supplementary Figure S22. Phylogenetic distribution of Spike H655Y inherited mutation, observed in Gamma and Omicron lineages.

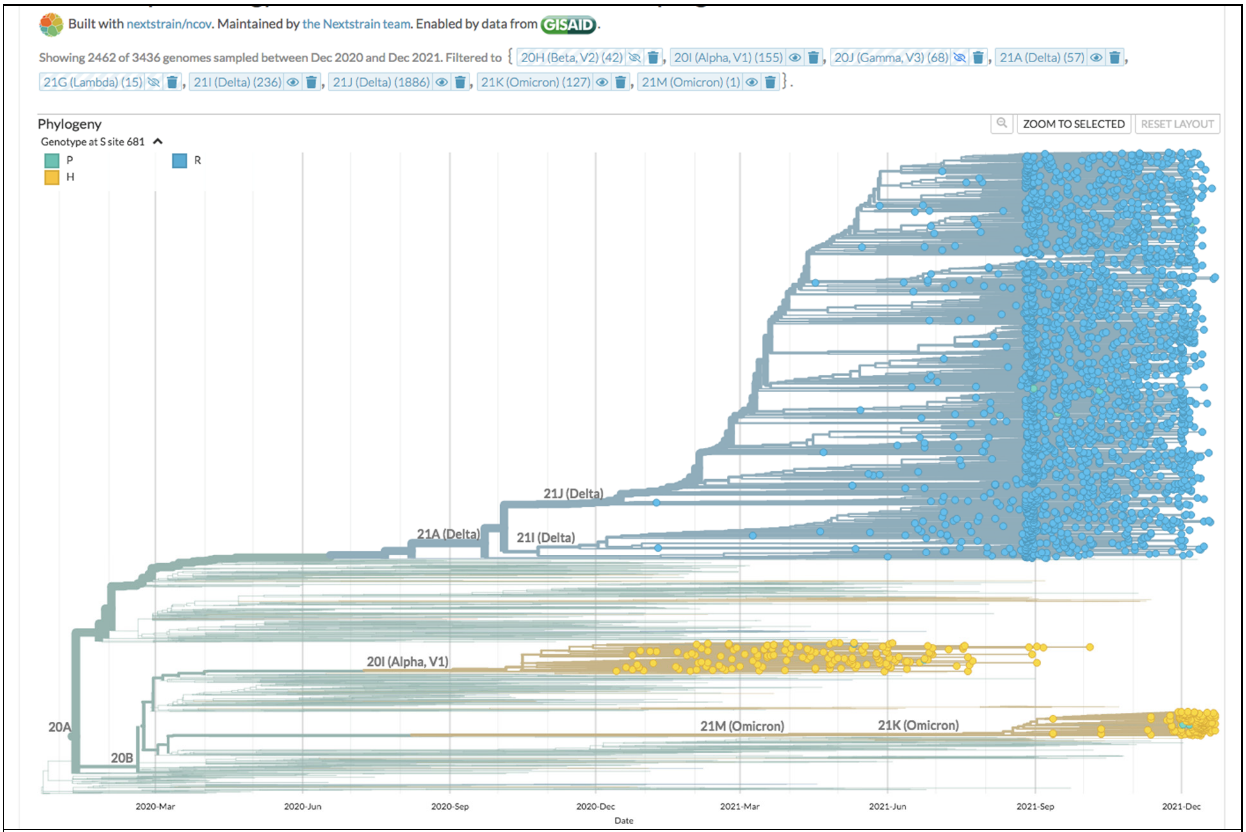

Supplementary Figure S23. Phylogenetic distribution of Spike P681H recurrent mutation, observed in Alpha and Omicron lineages.

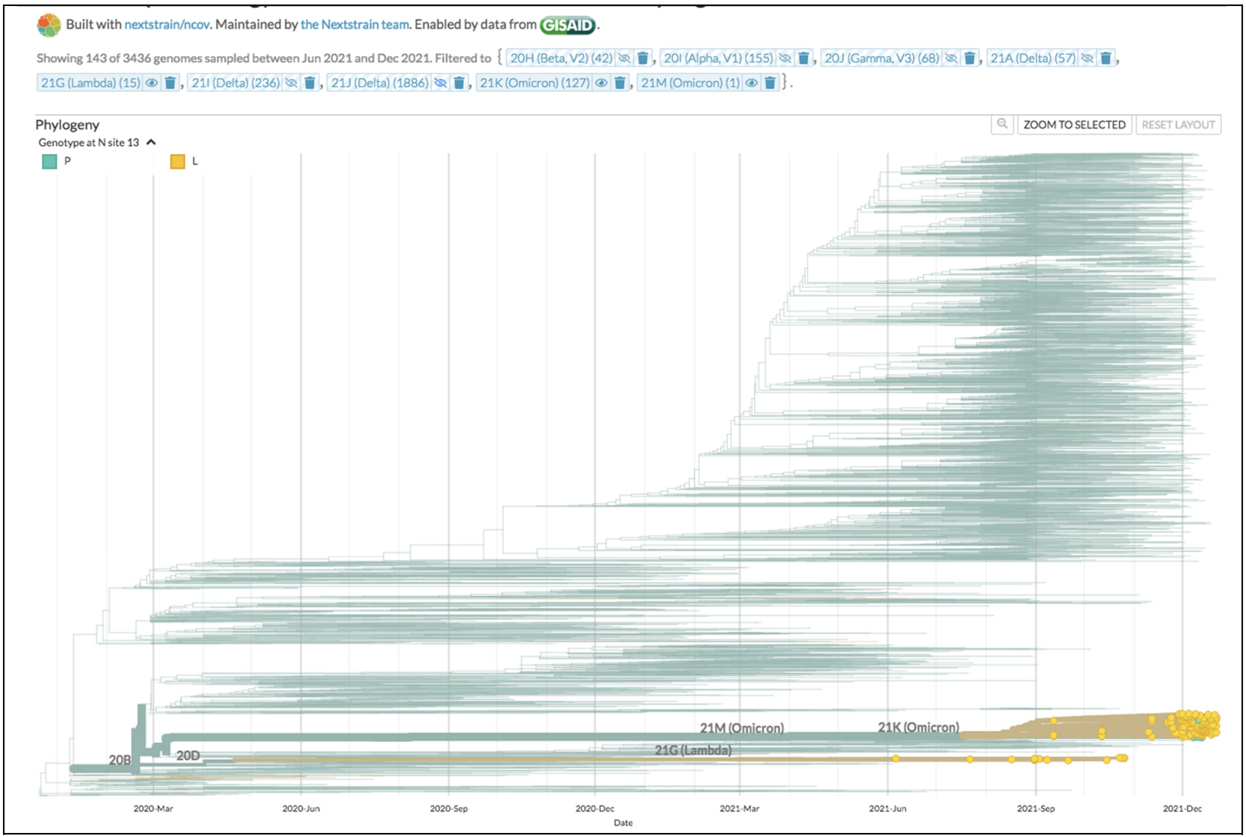

Supplementary Figure S24. Phylogenetic distribution of Nucleocapsid P13L recurrent mutation, observed in Lambda and Omicron lineages.

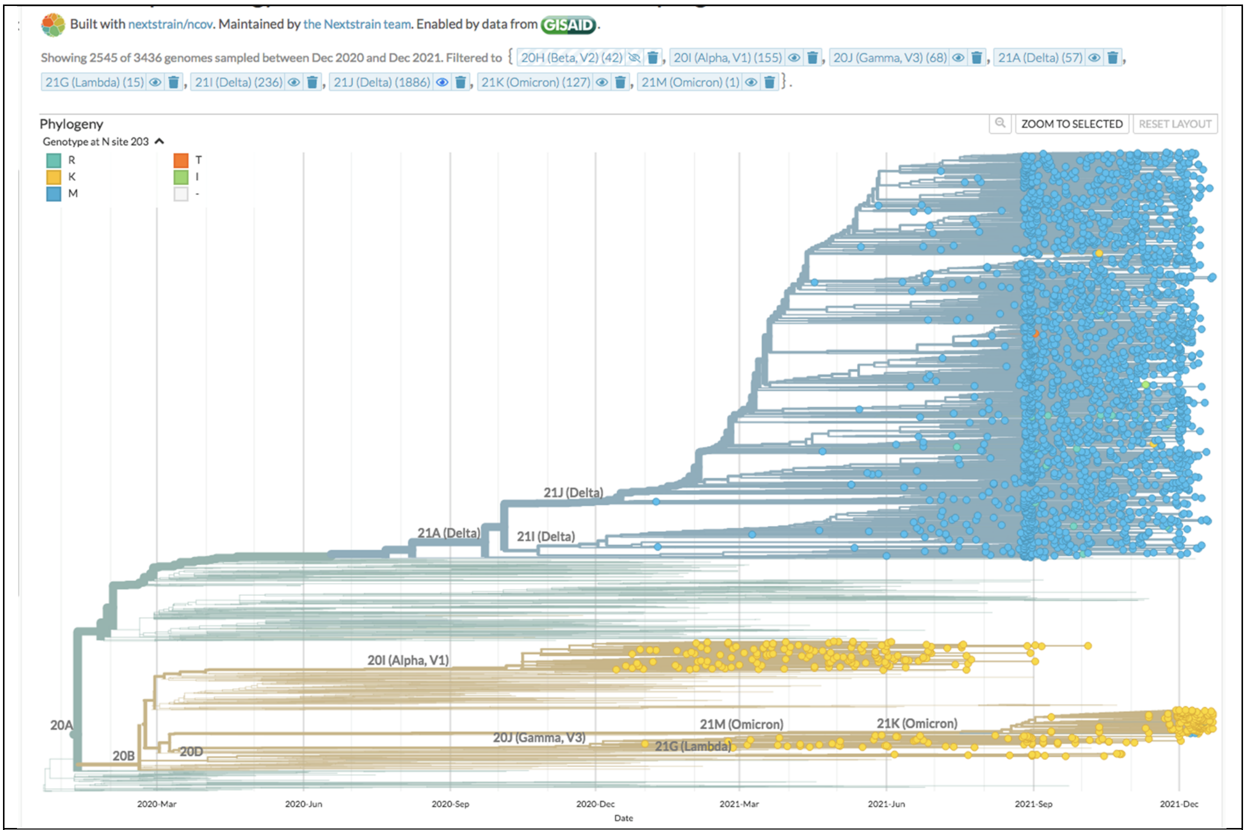

Supplementary Figure S25. Phylogenetic distribution of Nucleocapsid R203K inherited mutation, observed in Alpha, Gamma, Lambda and Omicron lineages.

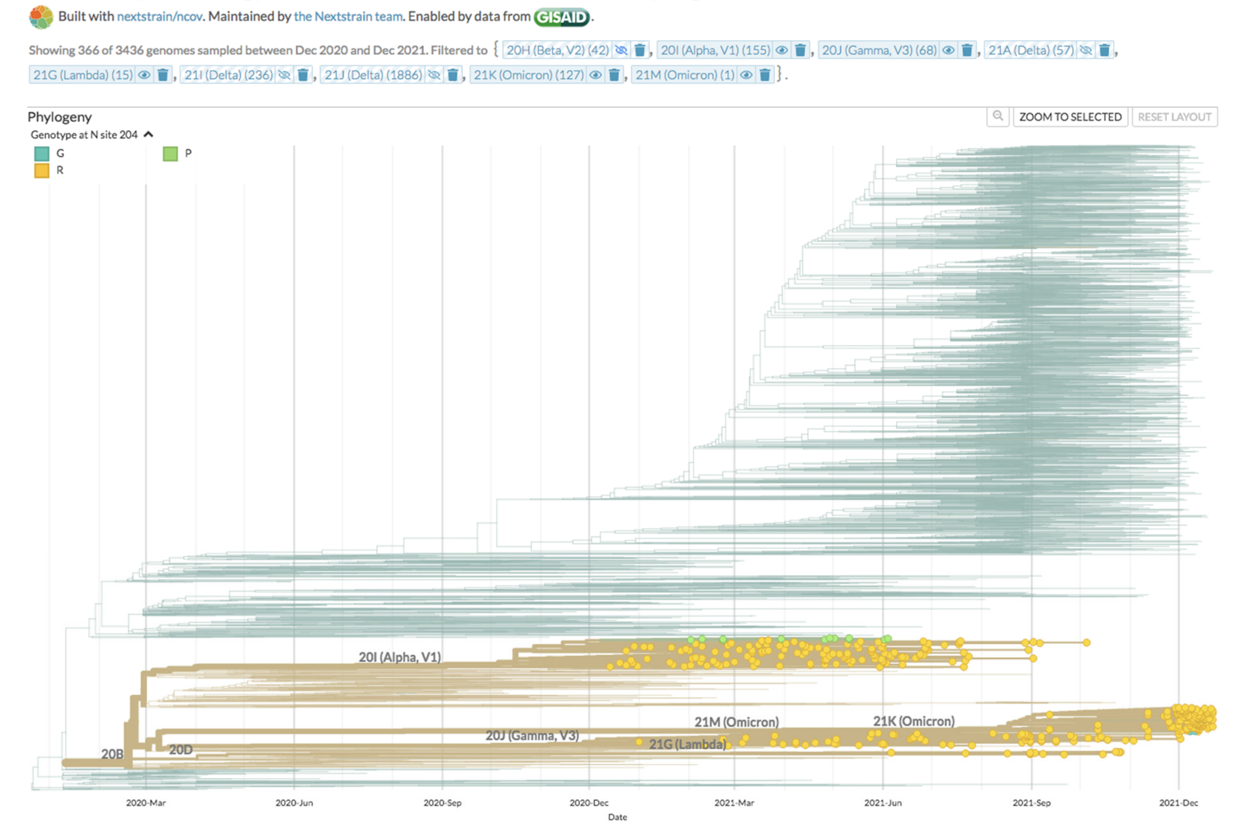

Supplementary Figure S26. Phylogenetic distribution of Nucleocapsid G204R inherited mutation, observed in Alpha, Gamma, Lambda and Omicron lineages.

Bibliography

1. Owen, D.R.; Allerton, C.M.N.; Anderson, A.S.; Aschenbrenner, L.; Avery, M.; Berritt, S.; Boras, B.; Cardin, R.D.; Carlo, A.; Coffman, K.J.; et al. An Oral SARS-CoV-2 Mpro Inhibitor Clinical Candidate for the Treatment of COVID-19. *Science* **2021**, *374*, 1586–1593, doi:10.1126/science.abl4784.
2. Kneller, D.W.; Phillips, G.; O'Neill, H.M.; Jedrzejczak, R.; Stols, L.; Langan, P.; Joachimiak, A.; Coates, L.; Kovalevsky, A. Structural Plasticity of SARS-CoV-2 3CL Mpro Active Site Cavity Revealed by Room Temperature X-Ray Crystallography. *Nat Commun* **2020**, *11*, 3202, doi:10.1038/s41467-020-16954-7.
3. Durdagi, S.; Dağ, Ç.; Dogan, B.; Yigin, M.; Avsar, T.; Buyukdag, C.; Erol, I.; Ertem, F.B.; Calis, S.; Yildirim, G.; et al. Near-Physiological-Temperature Serial Crystallography Reveals Conformations of SARS-CoV-2 Main Protease Active Site for Improved Drug Repurposing. *Structure* **2021**, *29*, 1382-1396.e6, doi:10.1016/j.str.2021.07.007.
4. Anandakrishnan, R.; Aguilar, B.; Onufriev, A.V. H++ 3.0: Automating PK Prediction and the Preparation of Biomolecular Structures for Atomistic Molecular Modeling and Simulations. *Nucleic Acids Res* **2012**, *40*, W537-541, doi:10.1093/nar/gks375.
5. Case, D.A.; Cheatham, T.E.; Darden, T.; Gohlke, H.; Luo, R.; Merz, K.M.; Onufriev, A.; Simmerling, C.; Wang, B.; Woods, R.J. The Amber Biomolecular Simulation Programs. *J Comput Chem* **2005**, *26*, 1668–1688, doi:10.1002/jcc.20290.
6. Salomon-Ferrer, R.; Götz, A.W.; Poole, D.; Le Grand, S.; Walker, R.C. Routine Microsecond Molecular Dynamics Simulations with AMBER on GPUs. 2. Explicit Solvent Particle Mesh Ewald. *J Chem Theory Comput* **2013**, *9*, 3878–3888, doi:10.1021/ct400314y.

7. Roe, D.R.; Cheatham, T.E. PTRAJ and CPPTRAJ: Software for Processing and Analysis of Molecular Dynamics Trajectory Data. *J Chem Theory Comput* **2013**, *9*, 3084–3095, doi:10.1021/ct400341p.
8. Humphrey, W.; Dalke, A.; Schulten, K. VMD: Visual Molecular Dynamics. *J Mol Graph* **1996**, *14*, 33–38, 27–28, doi:10.1016/0263-7855(96)00018-5.
9. Jaroszewski, L.; Iyer, M.; Alisoltani, A.; Sedova, M.; Godzik, A. The Interplay of SARS-CoV-2 Evolution and Constraints Imposed by the Structure and Functionality of Its Proteins. *PLoS Comput Biol* **2021**, *17*, e1009147, doi:10.1371/journal.pcbi.1009147.
10. Cubuk, J.; Alston, J.J.; Incicco, J.J.; Singh, S.; Stuchell-Brereton, M.D.; Ward, M.D.; Zimmerman, M.I.; Vithani, N.; Griffith, D.; Wagoner, J.A.; et al. The SARS-CoV-2 Nucleocapsid Protein Is Dynamic, Disordered, and Phase Separates with RNA. *Nat Commun* **2021**, *12*, 1936, doi:10.1038/s41467-021-21953-3.
